# Regulatory inter-domain interactions influence Hsp70 recruitment to the DnaJB8 chaperone

**DOI:** 10.1038/s41467-021-21147-x

**Published:** 2021-02-11

**Authors:** Bryan D. Ryder, Irina Matlahov, Sofia Bali, Jaime Vaquer-Alicea, Patrick C. A. van der Wel, Lukasz A. Joachimiak

**Affiliations:** 1 Molecular Biophysics Graduate Program, University of Texas Southwestern Medical Center, Dallas, TX USA; 2 Center for Alzheimer’s and Neurodegenerative Diseases, University of Texas Southwestern Medical Center, Dallas, TX USA; 3 Department of Structural Biology, University of Pittsburgh School of Medicine, Pittsburgh, PA USA; 4 Zernike Institute for Advanced Materials, University of Groningen, Groningen, Netherlands; 5 Neuroscience Graduate Program, University of Texas Southwestern Medical Center, Dallas, TX USA; 6 Department of Biochemistry, University of Texas Southwestern Medical Center, Dallas, TX USA

**Keywords:** Protein folding, Chaperones, Molecular modelling, NMR spectroscopy

## Abstract

The Hsp40/Hsp70 chaperone families combine versatile folding capacity with high substrate specificity, which is mainly facilitated by Hsp40s. The structure and function of many Hsp40s remain poorly understood, particularly oligomeric Hsp40s that suppress protein aggregation. Here, we used a combination of biochemical and structural approaches to shed light on the domain interactions of the Hsp40 DnaJB8, and how they may influence recruitment of partner Hsp70s. We identify an interaction between the J-Domain (JD) and C-terminal domain (CTD) of DnaJB8 that sequesters the JD surface, preventing Hsp70 interaction. We propose a model for DnaJB8-Hsp70 recruitment, whereby the JD-CTD interaction of DnaJB8 acts as a reversible switch that can control the binding of Hsp70. These findings suggest that the evolutionarily conserved CTD of DnaJB8 is a regulatory element of chaperone activity in the proteostasis network.

## Abstract (web summary)

The Hsp70/Hsp40 system plays an important role in maintaining cellular proteostasis but so far it is not well understood how Hsp70 proteins are recruited to specific Hsp40 co-chaperones. Here, the authors combine biochemical and biophysical approaches to characterise the oligomeric mammalian Hsp40 DnaJB8. They identify an intra-oligomer DnaJB8 interaction between the N-terminal J-Domain and the C-terminal domain that occludes the J-Domain surface that binds Hsp70 and propose a model for DnaJB8-Hsp70 recruitment.

## Introduction

The cellular chaperone network needs to handle a diversity of protein substrates in numerous different (mis)folded states. This demands a combination of broad versatility and specificity in terms of substrate recognition, even though the central players 70 kDa heat-shock protein (Hsp70) and 90 kDa Hsp (Hsp90) are highly conserved. This apparent contradiction is resolved by the Hsp40 (DnaJ) family of proteins, which are chaperones that recruit and regulate the activity of Hsp70 chaperones in refolding misfolded proteins^1–4^. While the human Hsp70 family is highly conserved, the Hsp40 chaperone family encodes 47 diverse members, each with specialized functions in substrate recognition and presumed coordination with Hsp70^5–7^. DnaJ proteins feature a J-domain (JD), which binds to Hsp70s through a conserved electrostatic interaction to trigger ATP hydrolysis by the Hsp70^7–10^. This initiates a conformational rearrangement in the Hsp70 substrate-binding domain that helps capture the substrate for folding, refolding, or disaggregation^11^. When misfolded proteins cannot be refolded, some Hsp40s help direct them for degradation^12,13^.

In humans, Hsp40s function as monomers, dimers, or oligomers. Classical Hsp40 members assemble into homodimers or mixed-class J-protein complexes^14,15^ through conserved C-terminal motifs and bind unfolded substrates through conserved β-barrel C-terminal domains (CTD)s^16^. A subset of nonclassical Hsp40s, including DnaJB2, DnaJB6b, DnaJB7, and DnaJB8, have a domain architecture that is distinct from the classical dimeric DnaJ orthologs^15–19^. These Hsp40s retain the JD, but have distinct other domains including substantial differences in their CTD structures. Of these, the DnaJB8 and DnaJB6b proteins self-assemble in vitro and in vivo^17,20,21^. The role of their CTD remains unclear, as the literature suggests that it either drives oligomerization or mediates intramolecular contacts^17–20,22^. The oligomers’ structural and dynamic heterogeneity has greatly hindered efforts to study them, yielding for DnaJB6 limited-resolution cryogenic electron microscopy data^21^ or requiring invasive deletion mutations to gain structural insight into soluble mutant variants^17–19^.

Here we examine DnaJB8, which has been shown to be particularly effective at preventing polyglutamine (polyQ) deposition, even more so than the homologous DnaJB6b despite 63% sequence identity^17,20,22,23^. This indicates that their specific modes of activity are distinct in spite of their similarities in sequence and domain arrangement. Notably, unlike other chaperones that inhibit mutant Huntingtin aggregation^24^, DnaJB8 and DnaJB6b are thought to bind directly to polyQ elements and thus are active across the whole family of polyQ diseases^17,23^. The two proteins have different expression profiles, with DnaJB8 being highly expressed in testes, while DnaJB6b is ubiquitous, which in part explains the deeper knowledge available for the latter protein. While both DnaJB6b and DnaJB8 assemble into soluble oligomers^17,20,21,23^, DnaJB8 in particular displays a higher propensity to assemble^17^. Here, we applied a multidisciplinary approach to understand the architecture and dynamics of DnaJB8 in cells and in vitro. We used cross-linking mass spectrometry (XL–MS) to identify local intra-domain contacts and long-range contacts. Guided by modeling, we mutated aromatic residues to create a monomeric mutant that maintains the intramolecular domain contacts observed in oligomers in cells and in vitro. Solid-state NMR (ssNMR) probed the structural and dynamic order of the solvated oligomers, to reveal dramatic domain-specific differences in (dis)order and a lack of highly flexible regions. Electrostatic interactions control the JD transitioning between an ordered immobilized state and a more mobilized state, which we attribute to JD–CTD interactions that we reconstitute with isolated domains and detect in full-length protein. Finally, we demonstrate that the JD–CTD contacts regulate the recruitment of Hsp70, representing a built-in regulatory mechanism that controls the recruitment (and thus activation) of Hsp70.

## Results

### DnaJB8 domain interactions in a cellular context

**Fig. 1:**
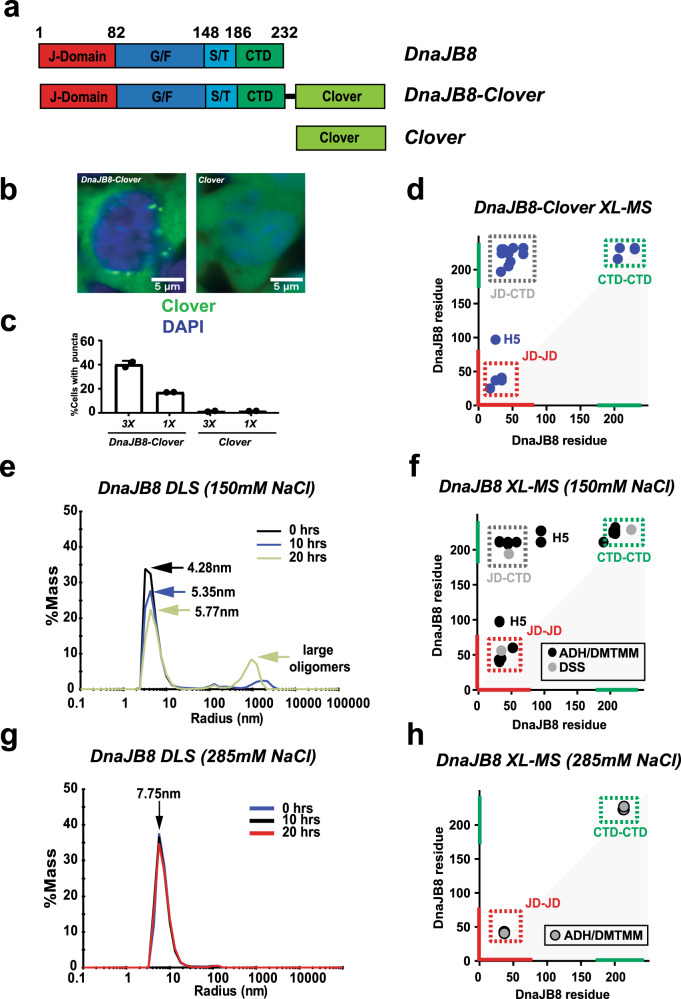
DnaJB8 architecture defined by domain-domain interactions. **a** Domain maps for DnaJB8 used in the in vitro experiments and the DnaJB–Clover and Clover constructs used in the mammalian cell experiments. DnaJB8 is colored according to domain annotation: JD (red), G/F rich (blue), S/T rich (cyan), and CTD (green). Clover is colored pale green. **b** Representative images of triplicate populations of 300,000 cells expressing DnaJB8–mClover3 (left) and mClover3 (right). Clover and DAPI fluorescence signals are shown in green and blue, respectively. Scale bar, 5 μm, is shown in white. **c** Quantification of DnaJB8–Clover and Clover puncta in high (3×) and low (1×) protein level expressing cell lines. In each analysis at least 2000 cells were counted by the CellProfiler software. Puncta were manually counted by two independent observers, with data reported as averages with standard deviation. **d** XL–MS contact map of DnaJB8–Clover cross-links identified using DMTMM and ADH. The axes are colored in red and green for JD and CTD, respectively. Cross-link pairs between JD–CTD, JD–JD, and CTD–CTD are shown in dashed boxes colored gray, red, and green, respectively. Contacts to helix 5 are denoted with H5. **e** Histogram of overall *R*_h_ of DnaJB8 in 1× PBS 150 mM NaCl from DLS at times 0 h (black), 10 h (blue), and 20 h (gold), with arrows indicating *R*_h_ peaks for each time point. Over time, there was a depletion in particle sizes <10 nm and an increase in particles ~100–1000 nm. **f** XL–MS contact map of DnaJB8 cross-links identified using DMTMM and ADH (black) and DSS (gray). The axes are colored in red and green for JD and CTD, respectively. Cross-link pairs between JD–CTD, JD–JD, and CTD–CTD are shown in a dashed box colored gray, red and green, respectively. Contacts to helix 5 are denoted with H5. **g** Histogram of overall *R*_h_ of DnaJB8 in 1× PBS 285 mM NaCl at times 0 h (blue), 10 h (black), and 20 h (red), with arrows indicating *R*_h_ peaks for each time point. Over time, there is no change in the species of particle sizes <10 nm and no appearance of particles ~100–1000 nm. **h** Contact map of DnaJB8 cross-links identified using ADH/DMTMM in the presence of 285 mM NaCl. The axes are colored in red and green for JD and CTD, respectively. JD–JD and CTD–CTD cross-links are shown in dashed boxes colored in red and green, respectively.

DnaJB8 encodes three domains C terminal to the JD (Fig. 1a): a glycine/phenylalanine (G/F)-rich domain (Fig. 1a, blue), a serine/threonine (S/T)-rich domain (Fig. 1a, cyan) and a CTD (Fig. 1a, green). Prior studies have highlighted the ability of DnaJB8 to assemble into oligomers, but little is known about DnaJB8 domain interactions in cells^17,22^. We expressed DnaJB8 fused to a green fluorescent protein (GFP) derivative mClover3 (herein, DnaJB8–Clover) in HEK293 cells (Fig. 1a). DnaJB8–Clover expression leads to the formation of fluorescent juxtanuclear puncta with an approximate maximum diameter of 1.0 μm (Fig. 1b) in 39.2 ± 3.1% of the cells (Fig. 1c), while Clover-alone expression yielded diffuse fluorescence (Fig. 1b) with few to no puncta (Fig. 1c; 0.44 ± 0.50%). The puncta observed in these cells indicate the presence of ordered aggregates, while the more uniformly dispersed signal is indicative of soluble oligomers and monomers. Decreasing the DnaJB8–Clover expression 3-fold as determined by western blot (Supplementary Fig. 1a) and fluorescence intensity (Supplementary Fig. 1b) yielded only a 2-fold decrease in the number of puncta (16.2 ± 0.08%; Fig. 1c). The frequency of puncta for Clover alone remained <1% in both experiments (Fig. 1c). Thus, even at reduced levels of expression DnaJB8 can form puncta in cells.

We next sought to characterize biochemical properties of DnaJB8–Clover expressed in mammalian cells. DnaJB8–Clover protein was purified using α-GFP nanobodies^25,26^ (Supplementary Fig. 1c). To gain insight into the topology of DnaJB8, we employed an XL–MS approach to define contacts between different domains^27–29^. Isolated DnaJB8–Clover was reacted with adipic acid dihydrazide (ADH) and 4-(4,6-dimethoxy-1,3,5-triazin-2-yl)-4-methyl-morpholinium chloride (DMTMM). ADH covalently links carboxylate–carboxylate contacts via a 6-carbon bridge, while DMTMM forms a direct covalent bond between lysine-carboxylate groups through dehydration^28^. Cross-linking treatment of the purified soluble DnaJB8–Clover species revealed predominantly monomers and dimers in the cells, with a trace of larger oligomers (Supplementary Fig. 1d). We identified 21 cross-links that parsed into three regions: JD–JD, CTD–CTD, and JD–CTD (Fig. 1d and Supplementary Data 1). The three local JD contacts (Fig. 1d, red box) are consistent with its experimental structure (Supplementary Fig. 1e). Local CTD contacts (Fig. 1d, green box) were also accompanied by inter-domain JD–CTD contacts that localize to helices 2 and 3 of JD (Fig. 1d, gray box). In addition, we identified a contact between the JD and a putative helix 5 (Fig. 1d, H5) of the G/F domain, as also recently identified in DnaJB6b^18,19^. Thus, soluble DnaJB8–Clover species isolated from mammalian cells reveal an array of inter-domain interactions, including contacts between the charge complementary JD and CTD.

### DnaJB8 domain contacts are preserved in vitro

For a more detailed understanding of DnaJB8 domain architecture in vitro, we produced recombinant DnaJB8 (see “Methods”). We first used dynamic light scattering (DLS) to monitor the hydrodynamic radius (*R*_h_) of DnaJB8 species over time. The scattering data reveals bona fide DnaJB8 sizes that begin as a small 4.28 ± 0.82 nm species with a small (<%1 by mass) contribution of larger species (>10 nm), but over time these small species shift to 5.35 ± 0.22 nm at 10 h and to 5.77 ± 0.43 nm after 20 h (Fig. 1e and Supplementary Fig. 1f). Over the time course, a fraction of the soluble small species converted into larger oligomers >10 nm (30.6% by mass) with an average *R*_h_ of 90 nm (Fig. 1e and Supplementary Fig. 1f). These findings are consistent with prior studies on DnaJB6b and DnaJB8 showing that they have the capacity to assemble into polydisperse soluble oligomers in vitro^17,20,21,23,30^.

Next, we aimed to better understand the topology of DnaJB8 in vitro using XL–MS, employing two parallel chemistries: disuccinimidyl suberate (DSS) and ADH/DMTMM on samples after a brief 30-min incubation. Consistent with the DLS data at early time points, we observe by sodium dodecyl sulfate-polyacrylamide gel electrophoresis (SDS-PAGE) a ladder of bands indicating the formation of covalent intermolecular contacts dominated by a dimer (Supplementary Fig. 1g). XL–MS analysis of these samples showed only three cross-links in the DSS condition (Fig. 1f and Supplementary Data 1). In contrast, the ADH/DMTMM analysis yielded 24 cross-links (Fig. 1f and Supplementary Data 1). Importantly, this XL–MS pattern persisted across the DLS time course (Supplementary Fig. 1h and Supplementary Data 1) and closely matches the pairs observed in the assemblies recovered from the mammalian cells including cross-links from both JD and CTD to H5 (Fig. 1d, H5). The JD cross-links are consistent with the structure of the domain (Supplementary Fig. 1i)^28^.

The CTD yielded eight cross-links (Fig. 1f and Supplementary Fig. 1h). Among these, the locally linked regions spanning E208-E211 and K223-K227 are central to the CTD and repeatedly react to peripheral sites. The third cluster of contacts linked the distal JD and CTD (Fig. 1f, JD–CTD and Supplementary Fig. 1h). Across experiments the sites on CTD that cross-link to the JD are mediated predominantly through acidic amino acids: E208, E209, E211, D212, but also K223, and K227. Conversely, across experiments the amino acids on the JD that cross-link to the CTD are predominantly lysines: K34, K44, K47, K60, and K61, but also E51 and E54 that localize to helix 3 (H3) and the loop prior to helix 4 (H4) (Supplementary Fig. 1j) and overlap the Hsp70-binding surface^10^. These XL–MS data identify an intricate network of electrostatic inter-domain interactions in both monomeric and oligomeric DnaJB8.

To further test the apparent role of electrostatically driven interactions, we used a higher ionic strength buffer in an analogous series of experiments. Using DLS we observed a defined species with a 7.75 ± 0.7 nm size in 285 mM NaCl (Fig. 1g and Supplementary Data 2), which is more expanded compared to species in 150 mM NaCl (Fig. 1e and Supplementary Data 2). XL–MS analysis recapitulates the short-range contacts within the JD and CTD domains, but the JD–CTD contacts were notably absent (Fig. 1h). To control for reactivity in each condition, we compared the frequency of ADH-driven singly reacted modifications, called monolinks. These data show nearly identical numbers of modifications, suggesting that the reactivity between these two conditions is nearly identical (Supplementary Fig. 1k). Thus, the disruption of electrostatically driven interactions is accompanied by changes in the domain architecture.

### JD–CTD interaction is mediated by electrostatic contacts

**Fig. 2:**
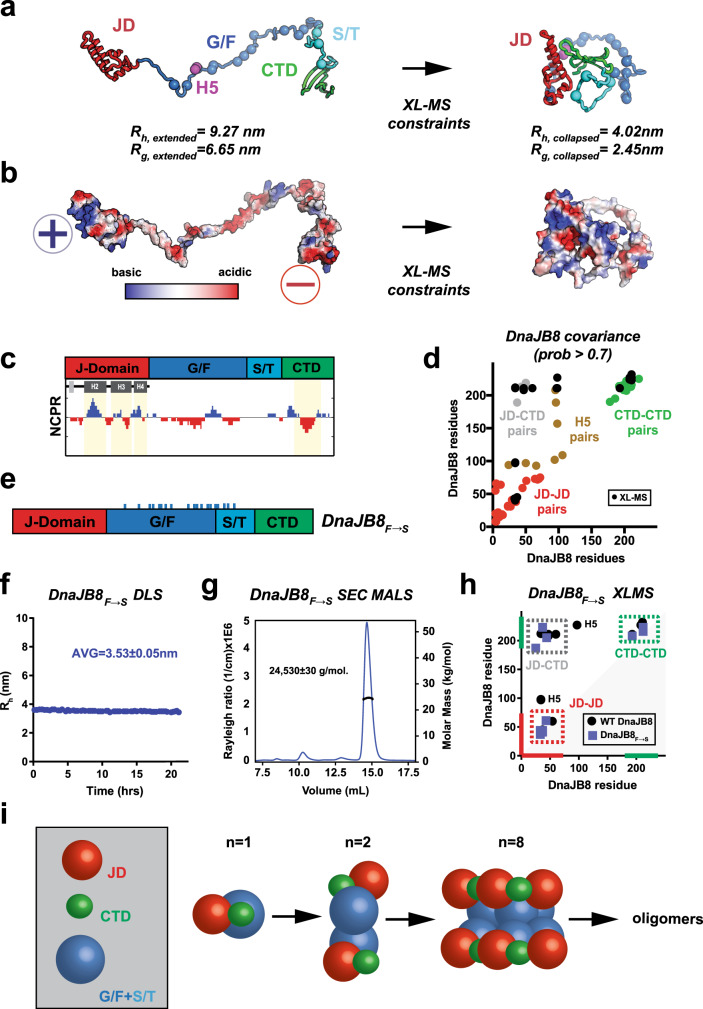
Model for the JD–CTD contacts in a DnaJB8 monomer. **a** XL–MS-based refinement of full-length expanded DnaJB8 monomer. Cartoon representation of DnaJB8 in fully expanded conformation (left) and collapsed conformation (right), colored by domain as in Fig. 1. Aromatic amino acids in the G/F and S/T domains are shown as spheres and colored according to the domain. Residues in helix 5 (H5) are shown as magenta spheres. Collapsed conformation model was selected from 1000 Rosetta ab initio generated models using a relax protocol. *R*_g_ and *R*_h_ values were calculated from the structural model in Rosetta and HYDROPRO, respectively. **b** Charge complementary surfaces on the JD and CTD mediate the interaction. Highly acidic potential is shown in red (− sign) and highly basic in blue (+ sign). **c** Net charge per residue (NCPR) distribution, defined as the average charge over a 10-residue window, highlights charge complementarity between basic and acidic residues on the JD and CTD, respectively (coloring as in Fig. 1). Helices in the JD with basic character are denoted as H2, H3, and H4. **d** GREMLIN sequence-based covariance analysis identified high confidence covarying amino acids on DnaJB8 that localize within the JD (red), within CTD (green), with H5 (brown), and across JD–CTD (gray). XL–MS links for full-length DnaJB8 (black dots) overlap with the covarying regions. Covarying positions localizing to amino acids in G/F domain are shown in brown and co-localize with XL–MS cross-links. **e** Domain map of the DnaJB8_F→S_ mutant, with mutated phenylalanine positions marked by cyan ticks. **f** DLS time course of the DnaJB8_F→S_ mutant. The average *R*_h_ was calculated to be 3.53 ± 0.05 nm. **g** SEC-MALS of CTD_170–232_ shows a single peak that was calculated to have a molar mass of 24,530 ± 30 g/mol consistent with a monomer. **h** XL–MS contact map showing ADH/DMTMM cross-links for WT DnaJB8 and DnaJB8_F→S_ mutant. The axes are colored in red and green for JD and CTD, respectively. Cross-link pairs between JD–CTD, JD–JD, and CTD–CTD are shown in a dashed box colored gray, red, and green, respectively. Contacts to helix 5 in WT DnaJB8 are denoted with H5. **i** Schematic of DnaJB8 species observed in solution based on DLS dimensions. Domains are shown as JD (red spheres), CTD (green spheres), and G/F + S/T (light blue spheres). The average *R*_h_ of DnaJB8_F→S_ (3.53 ± 0.05 nm) and DnaJB8 ab initio Rosetta model (4.02 nm) are assigned to the monomer. The *R*_h_ of WT DnaJB8 begins as a 4.28-nm species and grows to 5.77 nm over 20 h. Size and volume estimates from the structural models suggest DnaJB8 exists as small species ranging from a monomer to octamer likely dominated by a dimer and over time maturing into large oligomers.

To understand how the JD and CTD domains could be interacting, we used Rosetta modeling guided by XL–MS restraints. We built a starting model by combining the experimental structure of the JD (PDB ID: 2DMX) with an ab initio-derived model for CTD and the middle domains fully extended. The starting model was then collapsed by applying the JD–CTD cross-links as restraints (Fig. 2a and Supplementary Fig. 2a, b). The fully expanded monomer collapsed from a predicted *R*_h_ of 9.27 nm (*R*_g_, 6.65 nm) to 4.02 nm (*R*_g_, 2.45 nm) (Fig. 2a). Comparing these values to our DLS radii in 150 mM NaCl suggests that the dominant species are likely monomers and dimers. The DLS measurements in 285 mM NaCl are consistent with the initial expanded model with the JD–CTD contacts disengaged. Thus, our data support that DnaJB8 exists in solution as small-soluble species (4–6 nm), dominated by monomer/dimer but with the capacity to form larger oligomers over time, both in vitro and in vivo.

Guided by the constraints, the final model “docks” the JD onto the CTD placing a putative acidic surface on the CTD in contact with the basic surface on the JD (Fig. 2a and Supplementary Fig. 2b) and additionally bringing H5 in proximity to both the JD and CTD as similarly observed for DnaJB6b (Supplementary Fig. 2c)^18,19^. The CTD has proximal basic surfaces that flank its acidic surface, generating a characteristic alternating charge pattern that is inverted on the JD (Fig. 2c). Mapping sequence conservation onto the Rosetta-generated model, we find that these JD–CTD contacts are largely conserved (Supplementary Fig. 2d). In a coevolution analysis using the Gremlin algorithm^31–33^, we identified amino acid positions that covary. Not only did we observe many amino acid pairs that covary between the JD and CTD as well as H5, but our XL–MS pairs overlap with these covarying positions (Fig. 2d). The similarity between the predicted covarying contacts, conservation, and the XL–MS experimental contacts strengthens our DnaJB8 JD–CTD model, and suggests that XL–MS can detect functionally important interaction sites.

### JD–CTD contacts are present in monomeric DnaJB8

In our “collapsed” monomer structural model, the 17 phenylalanine residues in the G/F and S/T domains were predicted to be in part solvent exposed (Fig. 2a, spheres). We hypothesized that these aromatic residues may play a role in DnaJB8 assembly and engineered a mutant, in which all G/F- and S/T-region phenylalanine residues were mutated to serine residues (Fig. 2e, herein DnaJB8_F→S_). Using our DLS and XL–MS pipeline, we evaluated the assembly of DnaJB8_F→S_. By DLS, the DnaJB8_F→S_ mutant remained stable as a 3.53 ± 0.05 nm species over 21 h (Fig. 2f and Supplementary Data 2). SDS-PAGE of cross-linked DnaJB8_F→S_ also showed no intermolecular cross-links (Supplementary Fig. 2e). Size-exclusion chromatography multi-angle light-scattering (SEC-MALS) analysis on DnaJB8_F→S_ revealed it to be a monomer with a molecular weight of 24,530 ± 30 g/mol (Fig. 2g). These data support that phenylalanine residues in the G/F and S/T domains play a role in higher-order assembly. Next, we used XL–MS to test whether this DnaJB8_F→S_ monomer maintained the intramolecular JD and CTD contacts observed in wild-type (WT) DnaJB8 (Fig. 2h). Analysis of the cross-linked DnaJB8_F→S_ revealed identical local cross-links within JD and CTD and also detected three cross-links between the JD and CTD. Interestingly, in DnaJB8_F→S_, the H5 cross-links to JD were absent, consistent with the requirement of a phenylalanine in H5 for binding to the JD (Fig. 2h, H5). The presence of the JD–CTD cross-links in a monomeric mutant marks them to represent intramolecular JD–CTD interactions.

We can now use the experimental DLS radii with our structural models to more accurately infer the dimensions of the small soluble DnaJB8 species (Fig. 2i). At the start of the WT DnaJB8 DLS time course, we observed an initial population of polydisperse particles with an average radius of 4.28 ± 0.82 nm (Fig. 1e). The DnaJB8_F→S_ mutant showed a radius of 3.53 ± 0.05 nm with a very narrow monodisperse distribution, further supporting our model of a monomer “collapsed” by JD–CTD interactions. Based on a model proposed by Marsh and Forman-Kay^34^, we also estimate that a monomeric 232-residue DnaJB8 protein should have a size of 3.98 nm. These data support our analysis that WT DnaJB8 at first adopts primarily a monomer/dimer distribution that has the capacity to then assemble into large oligomers. In contrast, the larger DLS *R*_h_ values measured for DnaJB8 in 285 mM NaCl (Fig. 1g) are a result of the loss of the electrostatic JD–CTD contacts yielding a small oligomer mediated by aromatic contacts.

### ssNMR on DnaJB8 oligomers reveals regions of disorder and order

**Fig. 3:**
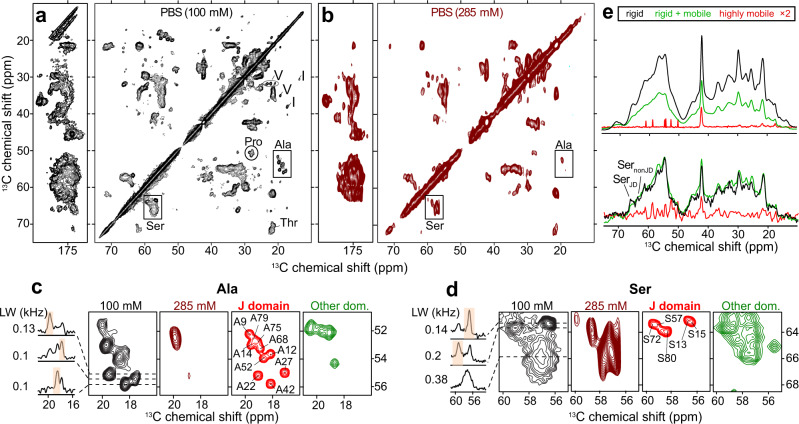
Solid-state NMR of DnaJB8 oligomers at physiological and high ionic strength. **a** 2D ^13^C-^13^C ssNMR spectrum of U-^13^C,^15^N-labeled DnaJB8 oligomers in PBS (100 mM NaCl), using 25 ms DARR mixing. **b** Corresponding 2D ssNMR spectrum in PBS with 285 mM NaCl. **c**, **d** Boxed Ala and Ser regions from panels (**a** and **b**). In PBS, the experimental Ala and Ser peak patterns (black) are well resolved and similar to those expected for folded JD in solution (red). At elevated ionic strength (brown) these narrow peaks are missing. Green spectra (right) represent simulated signals predicted for our models of the non-JD domains, shown with enhanced broadening reflecting the heterogeneity seen in the experiments. 1D spectra on far left show slices through the experimental 2D data, with selected peak widths (in kHz). **e** ^13^C 1D spectra, in PBS (top) and with 285 mM NaCl (bottom), that show rigid residues (black, CP), rigid and mobile residues (green, SPE), and only mobile residues (red, INEPT). See also text and Supplementary Fig. 3.

For additional insight into their molecular structure and dynamics, magic-angle-spinning (MAS) ssNMR was performed on the hydrated oligomers of U-^13^C-,^15^N-labeled DnaJB8. MAS ssNMR of hydrated protein assemblies allows for the site- and domain-specific detection of mobility and (secondary) structure, even in the presence of disorder and heterogeneity. 1D and 2D ssNMR spectra of the DnaJB8 oligomers feature many broad peaks, with linewidths up to 0.38 kHz, consistent with an oligomeric assembly displaying structural disorder (Fig. 3a and Supplementary Fig. 3a–d). However, strikingly, distinct subsets of narrow peaks are also detected, with linewidths of 0.1–0.2 kHz (Fig. 3c, d, left). These ssNMR experiments employ the cross-polarization (CP) technique, in which observable residues must be rigid or immobilized^35^. In insensitive nuclei enhancement by polarization transfer (INEPT)-based ssNMR, which is selective for highly dynamic segments, the oligomers show little signal^35–38^ (more below). Then, the observed narrow signals in CP spectra must originate from an immobilized, well-ordered subset of DnaJB8 residues. These narrow signals are from amino acid types^39^ in the JD, while the broad peaks are dominated by signals from residues common in other domains (Supplementary Table 1). The former also reflect mostly α-helical structure, while the latter are mostly random coil and β-sheet^40^. With known chemical shifts of the DnaJB8 JD in solution, we prepared a synthetic 2D spectrum (Supplementary Fig. 3b, red) that has a striking correspondence to the narrow ssNMR peaks (Supplementary Fig. 3b, black), such that we tentatively assign those to residues in H2 and H3 but also in H4. The 2D ^15^N-^13^Cα ssNMR spectrum showed a similar alignment between narrow peaks and JD signals in solution (Supplementary Fig. 3d). These CP-based 2D spectra also feature strong peaks from immobilized charged side chains (Lys, Arg, Asp, and Glu; Supplementary Fig. 3e, f), which is consistent with their involvement in salt bridge interactions predicted by the XL–MS analysis above.

In the absence of experimental solution NMR data for other domains, we predicted estimated spectra based on our structural models (Fig. 3c, d, green and Supplementary Fig. 3c)^41^. These peak patterns qualitatively resemble the broad signals in our 2D ssNMR data. A particular strength of MAS ssNMR of hydrated proteins is the ability to gauge local and global dynamics. Single-pulse excitation (SPE) and refocused INEPT spectra, which enhance the more dynamic parts of samples^37,38^, show surprisingly little evidence of flexible residues (Fig. 3e, top red). Indeed, the main INEPT signal (~42 p.p.m.) is just from solvent-exposed Lys side chains and lacks evidence of flexible protein regions (even from the G/F and S/T regions). Given that the 1D CP and SPE spectra (Fig. 3e, top) look similar, with higher signal intensities in the former, the different domains of the protein actually must have a similar degree of mobility and all be mostly immobilized, without flexible regions. Combined, the ssNMR data reveal oligomers that are heterogeneous in structure but lack extended flexible domains. In other words, the central G/F and ST domains are heterogenous, but also immobilized within the oligomers, consistent with the above-mentioned role of their Phe residues in driving oligomer assembly. Uniquely ordered are parts of the JD (residues in H2/H3/H4; Supplementary Fig. 3g, h), which show up as well folded and immobilized.

### Interaction sites from ssNMR

MAS ssNMR studies of DnaJB8 oligomers in phosphate-buffered saline (PBS) buffer with 285 mM NaCl (analogous to the studies above) are shown in Fig. 3b. The 2D spectrum reproduces the broad signals of the immobilized oligomers, but the narrow JD peaks are now strikingly absent. Comparing CP and SPE ssNMR spectra (Fig. 3e, bottom), there is an increase in overall mobility. Notably, no new “flexible” ssNMR signals were identified by INEPT ssNMR. We attribute the loss of JD signals in CP-based spectra to increased mobility due to disruption of long-range electrostatic interactions, while the lack of INEPT peaks tells us that the JD is still folded and partly immobilized by covalent attachment to the overall assembly. In other words, the JD is invisible due to intermediate timescale dynamics^35,42^. Since the broad signals from the other domains are preserved, it appears that the core architecture of the oligomers persists, consistent with aromatic and hydrophobic interactions.

### Isolated JD and CTD are folded and monomeric

**Fig. 4:**
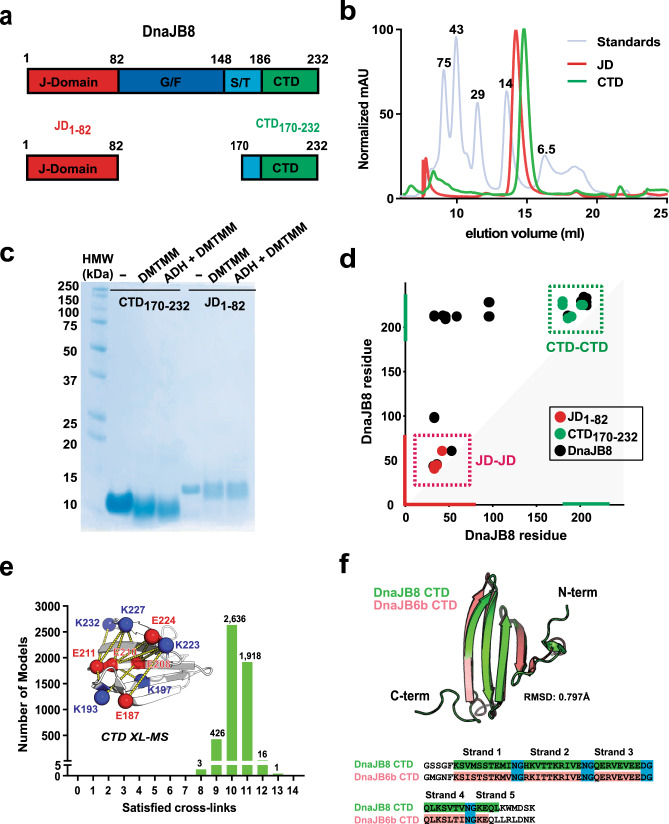
Isolated JD and CTD proteins are monomeric. **a** Cartoon schematic for the full-length DnaJB8 and domain fragments JD_1–82_ and CTD_170–232_. **b** Representative SEC profiles of JD_1–82_ (red), CTD_170–232_ (green), and LMW standards (blue). JD_1–82_ and CTD_170–232_ elute at apparent molecular weights of 14 and 6.5 kDa, respectively. **c** SDS-PAGE Coomassie gel of cross-linked JD_1–82_ and CTD_170–232_ reacted with either DMTMM only or DMTMM with ADH. This experiment was performed three independent times. **d** Contact map of ADH/DMTMM cross-links identified for JD_1–82_ (red), CTD_170–232_ (green), and full-length DnaJB8 (black). The axes are colored in red and green for JD and CTD, respectively. Cross-link pairs between JD–JD and CTD–CTD are shown in dashed boxes colored in red and green, respectively. **e** Histogram of the number of intra-domain cross-links that are consistent with cross-link chemistry geometry (“satisfied”) in the ensemble of 5000 models. One model satisfies 13 out of 14 possible cross-links identified in our experiments. Cross-links are mapped onto best matching CTD structural model (inset), shown in white cartoon representation. Sites of cross-link are shown as red or blue spheres, for D/E and K, respectively. Dashed yellow lines connect linked amino acid pairs. **f** Overlay of our DnaJB8 CTD model generated by ab initio ROSETTA (green) with the published DnaJB6bΔST CTD (salmon) (PDB ID: 6U3R). The CTD sequences of DNAJB8 and DNAJB6 are shown with each β-strand highlighted and conserved NG and DG turns in blue.

To further characterize the JD and CTD interaction, we produced isolated JD (herein JD_1–82_) and CTD (herein CTD_170–232_) (Fig. 4a). SEC analysis of JD_1–82_ and CTD_170–232_ revealed monodispersed peaks (Fig. 4b). SEC-MALS determined each domain to be monomeric with a molecular weight of 10,220 ± 220 and 8376 ± 14 g/mol for JD_1–82_ and CTD_170–232_, respectively (Supplementary Fig. 4a, b). Also, by DLS we measured the JD_1–82_ *R*_h_ to be 2.31 ± 0.13 nm and the CTD_170–232_ to be 1.71 ± 0.02 nm, with both stable over 15 h (Supplementary Fig. 4c and Supplementary Data 2). We again employed XL–MS to probe the individual domains and compare them to full-length protein. On an SDS-PAGE gel, the cross-linked JD_1–82_ and CTD_170–232_ remained monomeric following cross-linking (Fig. 4c). XL–MS analysis yielded four cross-links for JD_1–82_ and six cross-links for CTD_170–232_ (Fig. 4d and Supplementary Data 1). The identified cross-links revealed good agreement between the local domain cross-links observed in the full-length DnaJB8 and the isolated domains (Fig. 4d and Supplementary Fig. 4d).

We built an ensemble of models for the CTD_170–232_ using ab initio ROSETTA^40^. The calculated *R*_h_ for the structural ensemble was consistent with the DLS measurement of 1.7 nm (Supplementary Fig. 4g). The models formed a low contact order 5-stranded β-sheet topology and the *R*_h_ variation can be attributed to the more flexible termini (Supplementary Fig. 4g, inset). Circular dichroism on the CTD sample yields spectra consistent with a predominantly β-sheet content, as predicted by our model (Supplementary Fig. 4e, f). We mapped the 14 CTD-derived cross-links from across experiments onto the monomeric ensemble, finding that a majority of structures explain 10–11 cross-links, but only a single model explains 13 of 14 (Fig. 4e). These cross-link pairs map onto each face of the β-sheet and the distances are compatible with the geometry of the cross-linking chemistry. The cross-links that fall outside the distance cutoff localize to the more dynamic carboxy terminus of CTD (Fig. 4e and Supplementary Fig. 4g, inset) at positions K227 and K223. The CTD topology is defined by four β-turns stabilized by conserved asparagine/aspartate-glycine sequences (N/DG) and overlays well with the DnaJB6b CTD (Fig. 4f)^18,19^. Thus, our data support that both the JD_1–82_ and CTD_170–232_ domains are folded, monomeric, and do not have intrinsic assembly properties.

### Electrostatics drive JD interaction with CTD

**Fig. 5:**
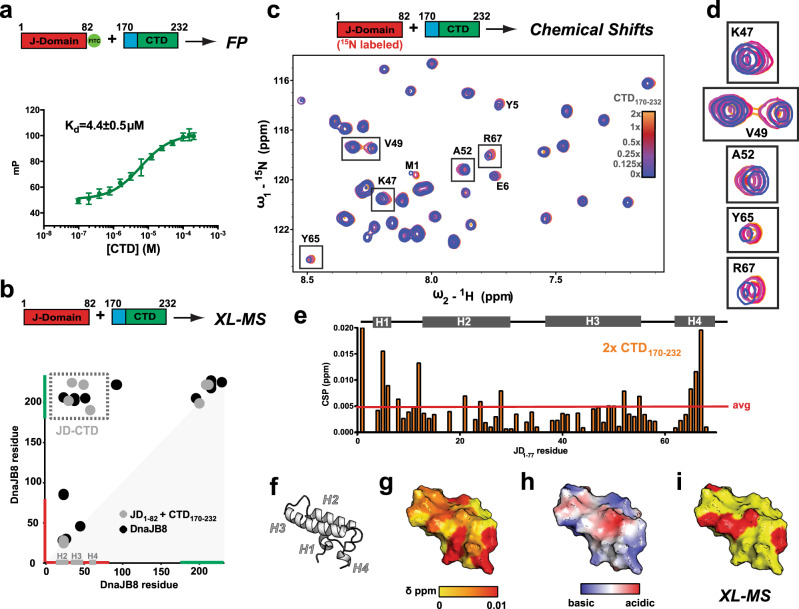
JD and CTD interact through charge complementary surfaces. **a** Schematic of the JD_1–82_-FITC (FITC dye is shown as a green circle) and CTD_170–232_ constructs used in fluorescence polarization (FP) experiments. FP titration measuringthe interaction between JD_1–82_–FITC and a concentration range of unlabeled CTD_170–232_. FP experiments were performed in triplicate and shown as averages with standard deviation. **b** Schematic of the JD_1–82_ and CTD_170–232_ constructs used in the XL–MS experiments. Contact map of ADH/DMTMM cross-links identified from an incubated JD_1–82_ and CTD_170–232_ sample (gray) and full-length DnaJB8 (black). The axes are colored in red and green for JD and CTD, respectively. H2, H3, and H4 are shown in gray on the *x*-axis. Cross-link pairs between JD–CTD are shown in a dashed box colored in gray. **c** Schematic for the solution NMR chemical shift experiment with U-^15^N JD titrated with unlabeled CTD. HSQC solution NMR spectrum of 50 μM ^15^N-labeled JD_1–82_ against a titration of CTD_170–232_: 0× (blue), 0.125× (purple), 0.25× (magenta), 0.5× (pink), 1× (red), and 2× (orange). DnaJB8 JD peak assignments were transferred from deposited data (BMRB: 11417). **d** Insets of peaks in H3 and H4 with highest observed chemical shifts: K47, V49, A52, Y65, and R67. Coloring as in panel (**c**). **e** Histogram of chemical shift perturbations (CSP) from 2× CTD experiment by residue. Average CSP of ~0.005 p.p.m. is denoted by the red line (excludes prolines). **f** DnaJB8 JD structure illustrating the locations of all helices (PDB ID: 2DMX). **g** Mapping CSP values onto the DnaJB8 JD structure, shown in surface representation and colored according to Δδ from low (0.0 p.p.m.) in yellow to high (red; 0.01 p.p.m.). **h** Electrostatic potential mapped onto DnaJB8 JD structure shown in surface representation. Highly acidic potential is shown in red and highly basic in blue. **i** JD surface structure (yellow) with residues that cross-link to the CTD are shown in red.

In our experiments on the full-length DnaJB8 oligomers, we observed that the JD and CTD interact through complementary electrostatic surfaces. We further probed this interaction by mixing the individual JD_1–82_ and CTD_170–232_ domains in vitro (Fig. 5a). We incubated flourescein (FITC)-labeled JD_1–82_ with a series of CTD_170–232_ concentrations and measured binding affinity using a fluorescence polarization (FP) assay. The resulting binding curve revealed that the JD_1–82_ binds to the CTD_170–232_ with 4.4 ± 0.5 μM affinity (Fig. 5a, bottom), which is consistent across technical replicates (Supplementary Fig. 5a; 6.21 ± 0.94 and 4.43 ± 0.55 μM), suggesting that this interaction is in the low micromolar range. JD_1–82_ and CTD_170–232_ domains were mixed together to form the complex and analyzed using XL–MS. We identified six local cross-link pairs consistent with pairs observed in full-length DnaJB8 and the isolated JD_1–82_ and CTD_170–232_ samples (Fig. 5b). Importantly, we also reconstitute four intermolecular contacts between the JD_1–82_ and CTD_170–232_ observed in full-length DnaJB8 experiments. However, an increased variance in the cross-link profile may indicate that the missing proximal sequences help define the proper architecture of the full-length DnaJB8 oligomers. Solution NMR-based chemical shift perturbation mapping was used to identify the JD_1–82_ surface that interacts with the CTD_170–232_ (Fig. 5 and Supplementary Fig. 5b, c). Titration of increasing amounts of unlabeled CTD_170–232_ into 50 μM ^15^N-labeled JD_1–82_ produced fast-exchanging concentration-dependent chemical shift perturbations in a specific subset of peaks (Fig. 5d, e); 17 peaks were perturbed (>0.005 p.p.m.). Among these perturbed peaks, nine residues are found along the face of H3 and H4 (Fig. 5f–h). In addition, three N-terminal residues with perturbed peaks were found along this same surface (Fig. 5f–h). These positions correlate with the same surface where we observed cross-links between the JD and CTD in full-length DnaJB8 (Fig. 5i), but also with the regions identified by ssNMR (Supplementary Fig. 3g, h). Other residues that show perturbations are basic residues on H2 that with the perturbations on H3 contribute to the surface that is coincident with the HspA1A binding face, consistent with ssNMR (Supplementary Fig. 3g, h). While a few other hydrophobic residues also show strong perturbations, all are in close proximity to charged residues along each helix. Given the small size of the JD_1–82_, it is likely that residues in the core behind the basic surface involved in the interaction experience changes in chemical shift.

### JD–CTD interaction competes with Hsp70 binding

**Fig. 6:**
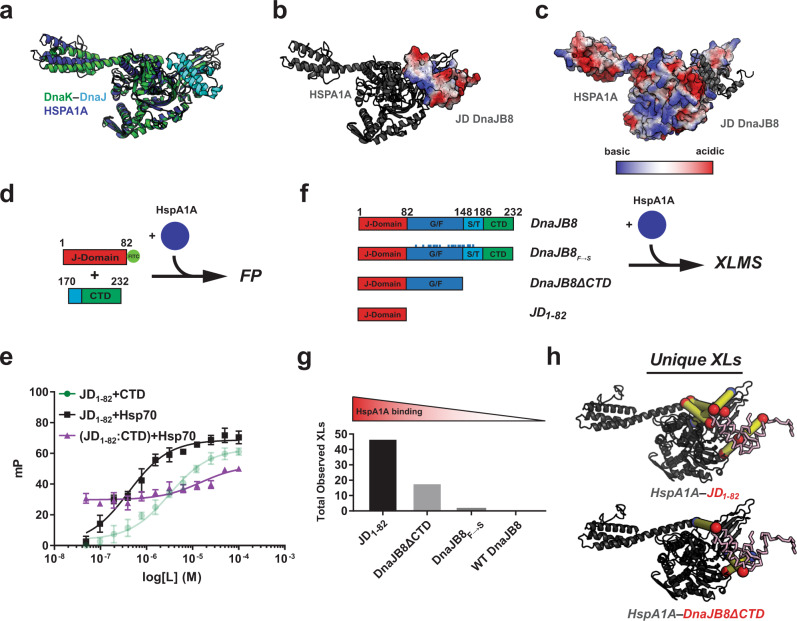
CTD and HspA1A compete for the same basic binding surface on DnaJB8 JD. **a** Structural superposition of a representative HspA1A structural homology model (blue) with a crystal structure of DnaK–DnaJ (green and cyan, respectively; PDB ID: 5NRO) shows good agreement. **b**, **c** Electrostatic surface potential of DnaJB8 JD docked into the JD binding site on HspA1A (shown in black cartoon representation). A basic surface on helix 2 docks onto the HspA1A surface. Electrostatic surface potential of HspA1A with docked DnaJB8 JD in black cartoon representation. The HspA1A surface presents an acidic face that complements the basic DnaJB8 JD surface. Highly acidic potential is shown in red and highly basic is shown in blue. **d** Experimental workflow used to determine competition between Hsp70 and CTD_170–232_ for JD_1–82_-FITC binding (dye shown as a green circle). **e** FP binding curves measuring affinity between fluorescent JD and added CTD (pale green) or added Hsp70 (black). Preincubation with CTD followed by the addition of Hsp70 (purple) shows a delay in binding consistent with a competitive binding model. FP experiments were performed in triplicate and shown as averages with standard deviation. **f** XL–MS-based experimental workflow used to determine the contribution of CTD to regulate JD binding to Hsp70. WT DnaJB8, DnaJB8ΔCTD, DnaJB8_F→S_, and JD_1–82_ DnaJB8 variants were used to form complexes with HspA1A. **g** Summary of total intermolecular cross-links identified across three XL–MS experiments between the JD and HspA1A for four complexes: JD_1–82_–HspA1A, DnaJB8ΔCTD–HspA1A, WT DnaJB8–HspA1A, and DnaJB8_F→S_–HspA1A. **h** Unique intermolecular cross-links identified across three datasets in the JD_1–82_–HspA1A and DnaJB8ΔCTD–HspA1A complexes mapped onto the JD–HspA1A model. JD is shown in pink ribbon representation and HspA1A in black cartoon representation. Sites of cross-link are shown as red or blue spheres for aspartic/glutamic acid and lysine, respectively. Yellow lines connect linked amino acid pairs.

The recent X-ray structure of the DnaK–DnaJ complex revealed a conserved charge-based interaction between the basic surfaces on the JD of DnaJ and an acidic surface on DnaK^10^. Using this complex as a template, we modeled the binding interface of the human Hsp70 (HspA1A)^43^ and the JD of DnaJB8^44^ (Fig. 6a–c). The basic surface on the DnaJB8 JD (Fig. 6b) contacts the conserved acidic surface on HspA1A (Fig. 6c and Supplementary Fig. 6a, b). Thus, conserved electrostatic contacts are likely to play a key role in the interaction between Hsp70 and Hsp40.

The conserved HspA1A–JD electrostatic contacts (Fig. 6b, c) that overlap with the JD–CTD contact sites lead us to hypothesize that the observed JD–CTD interactions could interfere with Hsp70 binding. To test this hypothesis, we employed a competition experiment leveraging our FP binding assay to discriminate the JD–CTD and JD–HspA1A complexes (Fig. 6d). We determine a 0.413 ± 0.057 μM affinity for the JD–HspA1A interaction, consistent with values in the literature^45^ and similar to the JD–CTD interaction (Fig. 6e, black and green, respectively). Due to the size difference between HspA1A (70 kDa) and the CTD (8.7 kDa), their respective complexes with tagged JD plateau at different polarization values (Supplementary Fig. 6c, black and green, respectively). Leveraging this difference, we designed a binding experiment to measure the competition of HspA1A and CTD binding to the JD. FITC-labeled JD was preincubated with 3 μM CTD, followed by a titration with HspA1A. The pre-titration FP signal was consistent with the formation of the JD–CTD complex, which persisted until HspA1A concentrations of 3.125 μM when the signal began to increase as HspA1A concentration exceeded the CTD concentration (Supplementary Fig. 6c, purple). We estimate that there is at least a 10-fold decrease in the apparent binding constant between JD–HspA1A when preincubated with CTD (Fig. 6e). To further test the inhibitory role of CTD on the recruitment of Hsp70, we used XL–MS to measure the frequency of HspA1A and JD contacts across a set of complexes formed between HspA1A and WT DnaJB8, JD_1–82_, DnaJB8_F→S_, and DnaJB8ΔCTD missing the CTD (Fig. 6f). Across three experiments, we detected no cross-links between the Hsp70 and the JD in WT DnaJB8 and only two in DnaJB8_F→S_ (Fig. 6g and Supplementary Data 1). In contrast, in the HspA1A–JD_1–82_ and HspA1A–DnaJB8ΔCTD complexes, we identified 47 and 14 total cross-links between the JD and HspA1A, respectively (Fig. 6g). All identified pairs are consistent with the structural model (Fig. 6h and Supplementary Fig. 6d). These data support that the robust JD–CTD engagement seen in WT DnaJB8 and even the monomeric DnaJB8_F→S_ (Supplementary Fig. 6e) prevents HspA1A interaction with the JD domain and deletion of the CTD releases the inhibitory effect (Fig. 6g). Thus, the DnaJB8 JD uses a basic surface to bind an internally encoded CTD via an acidic surface that directly inhibits HspA1A binding.

## Discussion

### Modeling the shape of DnaJB8

DnaJB8, like DnaJB6b, has the capacity to assemble into soluble oligomers. We used a combination of protein engineering, solution scattering data, and modeling to understand the shapes of DnaJB8 in the solution. Using our XL–MS data, we collapsed a DnaJB8 structural model around the JD–CTD interaction and thus obtained a structural model that fit the average *R*_h_ of the monomer measured by DLS. Based on the fold of this monomeric model, we hypothesized that aromatic amino acids in the central G/F and S/T domains would be exposed and thus could mediate self-assembly into oligomers. Indeed, mutagenesis of aromatic residues yielded a stable monomeric variant of DnaJB8 in agreement with our collapsed structural model with engaged JD–CTD contacts. This is further supported by a good agreement between the *R*_h_ of our collapsed structural model, DLS data, and values derived from the Marsh and Forman-Kay model^34^. An intriguing question relates to whether our models may also be applicable to DnaJB6b. At this time, a direct comparison is difficult given the known structural and functional differences of the proteins and the lack of analogous experimental data, especially on the larger oligomers of DnaJB6b. Our collective data highlight the power of our multipronged approach to derive the base unit of a DnaJB8 monomer, which employs exposure of aromatic residues to mediate assembly through nonpolar surfaces into larger oligomers.

### Functional role of the CTD in DnaJB8

We combined XL–MS and NMR in the solid and solution states to probe DnaJB8 inter-domain interactions. One of the most striking features was an interaction between the distal JD and CTD driven by electrostatics. This interaction was perturbed by the addition of salt, but maintained following mutagenesis of aromatic amino acids in the central domains. Since analysis of the isolated JD and CTD showed a reduced mutual association, there nonetheless is a distinct role for the intervening domains in the JD–CTD interaction. Our combined data show that the DnaJB8 S/T and G/F domains are not behaving as “flexible linkers”^18,19^ and that their aromatic residues are central in the homo-oligomerization process. On their own, both JD and CTD are surprisingly resistant to self-assembly. These findings are distinct from published reports on DnaJB6b, where the CTD appears to drive oligomerization, which may relate to sequence divergence in the six C-terminal CTD residues between DnaJB8 and DnaJB6b^18–20^. Nonetheless, our modeled CTD structure, featuring a pleated β-sheet topology absent of a hydrophobic core, is identical to its recently reported DnaJB6b counterpart^18,19^. Interestingly, outside inter-strand hydrogen bonding and polar side-chain contacts, it is not clear what forces stabilize this domain. This may explain the CTD heterogeneity (unlike the JD) seen by ssNMR. The CTD topology resembles the charged β-sheet surface on Hsp70 that is known to interact with the JD^10^. While our reconstitution of the JD–CTD interaction using isolated domains indicates that the CTD alone can bind the JD, we cannot exclude that helix 5 can contribute to this interaction to regulate Hsp70 function. It is worth noting that lysine residues in the DnaJB8 and DnaJB6b CTD can be acetylated and deacetylated (via histone deacetylases) to modify these proteins’ self-assembly and function, which may involve changes in the K-mediated JD interactions^17,22^. The CTD architecture is conserved in a broader subset of B family member Hsp40s^20^. We speculate that the CTD in these DnaJB family members similarly serves a regulatory role in which posttranslational modifications could alter the affinity for the JD, and thus indirectly alters oligomerization or Hsp70 recruitment.

### Implications for Hsp70 recruitment and substrate binding

**Fig. 7:**
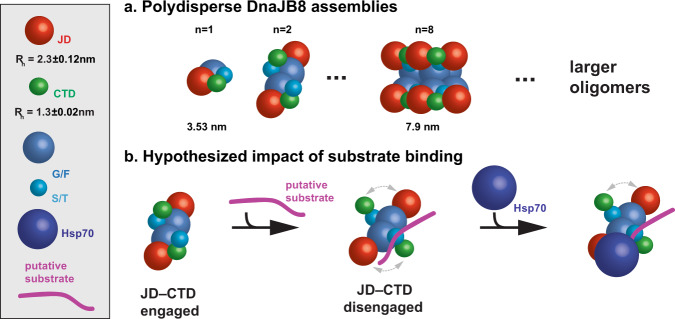
Proposed model for DnaJB8–HspA1A–substrate relationship. Schematic of proposed DnaJB8 model. Domains are shown as JD (red spheres), CTD (green spheres), G/F (blue spheres), S/T (light blue spheres), and also HspA1A (dark blue spheres) and substrate (purple line) are shown. DnaJB8 domain sizes are displayed scaled to the relative *R*_h_ values derived from DLS experiments (HspA1A not drawn to scale). **a** DnaJB8 forms a fundamental oligomeric species through aromatic contacts in the G/F and S/T domains ranging from monomer to octamer. **b** The JD–CTD engaged state, where the JD is stabilized by CTD and helix 5 (G/F) contacts, can form larger polydisperse oligomers (>100 nm). The JD–CTD disengaged state (bottom) is needed to engage with HspA1A. We illustrate our hypothesis where substrate binding may allosterically disrupt the JD–CTD interaction to allow the recruitment of HspA1A to the freed JD–CTD binding face, enabling subsequent handoff of the substrate to HspA1A.

Aside from suppressing protein aggregation on its own^17,20,22^, DnaJB8 also recruits Hsp70 for the processing of bound substrates. Our current findings hint at an intriguing possibility that autoinhibitory interactions of the Hsp70-binding JDs within the DnaJB8 oligomer could be involved in substrate-binding-coupled Hsp70 recruitment. In the non-stressed native state, DnaJB8 forms soluble oligomers in which the JD is engaged in electrostatic interactions and thus not available for Hsp70 binding as supported by our experiments (Figs. 6g and 7a). We hypothesize that substrate binding could allosterically disrupt the JD–CTD interaction, exposing the Hsp70-binding HPD motif of the JD (Fig. 7b). This would enable the recruitment of Hsp70 to the loaded DnaJB8 protein. Aromatics-driven oligomeric assembly of DnaJB8 may be related to the formation of liquid–liquid phase-separated assemblies in other proteins containing similar arrangements of phenylalanine residues^46^. We propose that the more hydrophobic elements of the G/F and S/T domains form the oligomer core, with the CTD and JD remaining relatively surface exposed. Thus, it may be possible to recruit Hsp70 to different DnaJB8 species. Our data on the DnaJB8_F→S_ mutant illustrate that the JD–CTD interaction exists in the monomeric base unit suggesting that this interaction is present across the polydisperse distribution of DnaJB8 species. Although we as yet lack detailed information supporting a substrate-triggered modulation of the JD–CTD interaction, our results offer some hints toward a possible molecular mechanism for such a coupling. In in vivo and in vitro XL–MS experiments, negatively charged residues in helix 5 in the G/F domain interact with both the JD and CTD (Fig. 1). We also saw a change in JD–CTD affinity in the absence of the central domains (Fig. 5). Finally, other studies on DnaJB6b have identified the S/T domains as substrate-binding domains^17,22,23^. Future mechanistic and structural studies on DnaJB8 and other complex chaperones including DnaJB6b and their interactions with substrates will reveal the interplay between oligomer dynamics, posttranslational modifications, substrate binding, and recruitment of Hsp70.

## Methods

### Sequence and structural analysis of DnaJB8, DnaJB6b, and HspA1A

Analysis of protein sequences (including the net charge per residue) was performed using Local CIDER^47^. An ensemble of 1000 HspA1A homology models was produced using ab initio Rosetta using the DnaK (PDB ID: 5NRO) conformation as a template^10^. Briefly, the HspA1A sequence was aligned to the DnaK sequence to identify regions with loop insertions and deletions. The HspA1A fragment library was produced using the fragment picker. The lowest scoring model was used to produce a model of the complex between HspA1A and the JD of DnaJB8. The structural images were produced using PyMOL.

### Cell biological and biochemical analysis of DnaJB8–Clover cell lines

The human DnaJB8 protein-coding sequence was cloned using Gibson assembly into a modified FM5 lentiviral expression plasmid^48^, in which the UbC promoter was replaced by a CMV promoter, the linker sequence was replaced by “GSAGSAAGSGEF,” and the YFP was replaced by mClover3. The primers used are listed in Supplementary Table 2. The resulting gene produced a DnaJB8–mClover3 fusion protein. In parallel, we produced a construct that expresses the fluorescent protein (mClover3) but lacks DnaJB8. Both plasmids we separately co-transfected into HEK293T cells along with helper plasmids (pCMV-VSV-G and psPAX2) to produce lentivirus, which was harvested after 48 h and used to produce polyclonal cell lines that expressed either DnaJB8–mClover3 or mClover3. For cross-linking experiments, cells from a confluent 10-cm^2^ cell culture dish were pelleted and lysed using an insulin syringe in 1× PBS with 1 mM dithiothreitol (DTT), 1 mM phenylmethylsulfonyl fluoride (PMSF), 1× EDTA-free Protease Inhibitor Cocktail (Roche), and 1% digitonin. After spinning at 1000 × *g* for 10 min, the lysate was recovered and incubated with a polyhistidine-tagged anti-GFP nanobody (plasmid encoding the nanobody^26^ was a kind gift from Dr. Judith Frydman) for 2.5 h at 4 °C. Briefly, nanobody expression was induced in BL-21 (DE3) cells using 0.5 mM isopropyl β-d-1-thiogalactopyranoside (IPTG) at 37 °C for 4 h, purified using a HisPur^TM^ Ni-NTA Resin (Thermo Scientific), and sample purity was verified using SDS-PAGE. The purified nanobody samples were flash frozen in liquid nitrogen and stored in −80 °C. The nanobody and HEK293T cell lysate mix was then incubated with 25 μL HisPur^TM^ Ni-NTA Resin (Thermo Scientific) for 1 h at 4 °C for binding. The beads were washed five times with 300 μL 1× PBS. The buffer for each wash was removed after pulse spinning the beads via centrifugation. The beads were preincubated for 5 min at 37 °C and a final concentration of 57 mM ADH and 36 mM DMTMM were added to each sample. Following a 1-min incubation with chemical crosslinkers, the reaction was quenched with 1 mM ammonium bicarbonate. After another pulse spin to remove the buffer, the beads were resuspended in the elution buffer (8 M urea, 0.5 M imidazole, pH 7.5). After a final pulse spin, the supernatant was retained and analyzed by MS and western blot.

### Cross-linking reagents

All cross-linking reagents used are commercially available: ADH (Sigma-Aldrich), mixed light and deuterated ADH (ADH-h_8_/d_8_) (Creative Molecules), mixed light and deuterated DSS (DSS-h_12_/d_12_) (Creative Molecules) and DMTMM (Sigma-Aldrich). For all cross-linking experiments, stock solutions were made of each cross-linking reagent. ADH stock solutions were made at 100 mg/mL in 1× PBS pH 7.4 (Sigma-Aldrich). DMTMM (Sigma-Aldrich) was prepared at a 120 mg/mL concentration in 1× PBS pH 7. DSS stock solutions were made at a 25 mM concentration in Dimethyl Formamide (DMF).

### Cross-linking MS

The ex vivo purified DnaJB8 was dialyzed to remove excess imidazole, and transferred into 1× PBS pH 7.4 buffer. For the full-length DnaJB8 experiments, lyophilized DnaJB8 was resuspended in either 1× PBS (150 mM) or 1× PBS (285 mM) to a concentration of 100 μM. The JD_1–82_ and CTD_170–232_ constructs were purified into 1× PBS buffer, and were prepared for XL–MS experiments at 100 μM each. Two micromoles of HspA1A were dissolved in 1× PBS pH 7.4 buffer and mixed with either 40 μM DnaJB8, 40 μM JD_1–82_, 40 μM DnaJB8ΔCTD, and 40 μM DnaJB8_F→S_ for XL–MS experiments and performed in triplicate. All samples were incubated at 37 °C while shaking at 350 r.p.m. for 30 min. Final concentrations of 57 mM ADH-h_8_/d_8_ (Creative Molecules) and 36 mM DMTMM (Sigma-Aldrich) or 1mM DSS-h_12_/d_12_ (Creative Molecules) were added to the protein samples and incubated at 37 °C with shaking at 350 r.p.m. for 30 min. The reactions were quenched with 100 mM ammonium bicarbonate and incubated at 37 °C for 30 min. Samples were lyophilized and resuspended in 8 M urea. Samples were reduced with 2.5 mM tris(2-carboxyethyl)phosphine (TCEP) incubated at 37 °C for 30 min, followed by alkylation with 5 mM iodoacetimide for 30 min in the dark. Samples were diluted to 1 M urea using a stock of 50 mM ammonium bicarbonate and trypsin (Promega) was added at a 1:50 enzyme-to-substrate ratio and incubated overnight at 37 °C while shaking at 600 r.p.m. Two percent (v/v) formic acid was added to acidify the samples following overnight digestion. All samples were run on reverse-phase Sep-Pak tC18 cartridges (Waters) eluted in 50% acetonitrile with 0.1% formic acid. Ten microliters of the purified peptide fractions was injected for liquid Chromatography with tandem mass spectrometry analysis on an Eksigent 1D-NanoLC-Ultra HPLC system coupled to a Thermo Orbitrap Fusion Tribrid System. Peptides were separated on self-packed New Objective PicoFrit columns (11 cm × 0.075 mm ID) containing Magic C18 material (Michrom, 3 μm particle size, 200 Å pore size) at a flow rate of 300 nL/min using the following gradient: 0–5 min = 5% B, 5–95 min = 5–35% B, 95–97 min = 35–95% B, and 97–107 min = 95% B, where A = (water/acetonitrile/formic acid, 97:3:0.1) and B = (acetonitrile/water/formic acid, 97:3:0.1). The MS was operated in data-dependent mode by selecting the five most abundant precursor ions (*m*/*z* 350–1600, charge state 3+ and above) from a preview scan and subjecting them to collision-induced dissociation (normalized collision energy = 35%, 30 ms activation). Fragment ions were detected at low resolution in the linear ion trap. Dynamic exclusion was enabled (repeat count 1, exclusion duration 30 s).

### Analysis of MS results

All MS experiments were carried out on an Orbitrap Fusion Lumos Tribrid instrument available through the UTSW proteomics core facility. Each Thermo.raw file was converted to.mzXML format for analysis using an in-house installation of xQuest^49^. Score thresholds were set through xProphet^49^, which uses a target/decoy model. The search parameters were set as follows. For grouping light and heavy scans (hydrazide cross-links only): precursor mass difference for isotope-labeled hydrazides = 8.05021 Da for ADH-h_8_/d_8_; maximum retention time difference for light/heavy pairs = 2.5 min. Maximum number of missed cleavages = 2, peptide length = 5–50 residues, fixed modifications = carbamidomethyl-Cys (mass shift = 57.02146 Da), mass shift of light cross-linker = 138.09055, mass shift of monolinks = 156.1011 Da, MS^1^ tolerance = 15 p.p.m., and MS^2^ tolerance = 0.2 Da for common ions and 0.3 Da for cross-link ions; search in enumeration mode. For zero-length cross-link search: maximum number of missed cleavages = 2, peptide length = 5–50 residues, fixed modifications carbamidomethyl-Cys (mass shift = 57.02146 Da), mass shift of cross-linker = −18.010595 Da, no monolink mass specified, MS^1^ tolerance = 15 p.p.m., and MS^2^ tolerance = 0.2 Da for common ions and 0.3 Da for cross-link ions; search in enumeration mode. For grouping heavy and light scans (succinimide cross-links only): precursor mass difference for isotope-labeled succinimides = 12.07573 Da for DSS-h_12_/d_12_; maximum retention time difference for light/heavy pairs = 2.5 min. Maximum number of missed cleavages (excluding the cross-linking site) = 2, peptide length = 5–50 aa, fixed modifications = carbamidomethyl-Cys (mass shift = 57.021460 Da), mass shift of the light crosslinker = 138.068080 Da, mass shift of mono-links = 156.078644 and 155.096428 Da, MS1 tolerance = 10 ppm, MS2 tolerance = 0.2 Da for common ions and 0.3 Da for cross-link ions, search in enumeration mode. The false discovery rates of all in vitro experiments range from 0.05 to 0.33.

### Western blot analysis

Ten microliter aliquots of the HEK control, Clover, and DnaJB8–Clover cell lines were removed from the elution and loaded onto a 4–12% Bis-Tris SDS-PAGE gel for western blotting. Upon running the gel to completion, the gel transferred onto a transfer membrane soaked in Novoblot transfer buffer. Following the transfer, the membrane was soaked in milk blocking buffer for 1 h at room temperature. For immunolabelling, we added 1:2000 dilution of polyclonal anti-GFP (rabbit) (Rockland; 600-401-215; 35460) or anti-DnaJB8 (rabbit) (Abcam; ab235546; GR3229943-2) in milk and incubated the membrane shaking at room temperature for 2 h. For imaging loading standards, we used monoclonal anti-GAPDH (mouse) (Novus Biologicals; NB300-221; 082219). The primary antibody solution was dumped and the membrane washed three times for 10 min each with 1× TBST before adding the polyclonal anti-rabbit IgG peroxidase (Cytiva; NA9340V; 16908235) or polyclonal anti-mouse IgG peroxidase (Cytiva; NA931V; 17089105) at a 1:5000 dilution in milk. The membrane was incubated with a secondary antibody at room temperature for 1 h before removing the antibody solution. The membrane was washed three times in 5-min intervals with 1× TBST and finally one 5-min wash with 1× TBS. The membrane was soaked in 1 mL of Luminol enhancer and peroxide solution for 1 min before imaging.

### In cell analysis of DnaJB8–Clover and Clover cell lines

HEK293T cells were treated with 1× and 3× amounts of lentivirus expressing either DnaJB8–mClover3 or mClover alone were plated at 300,000 cells per well in media (10% FBS, 1% Pen/Strep, 1% GlutaMax in Dulbecco’s modified Eagle’s medium) in a 6-well glass bottom plate (Cellvis, P06-1.5-N). After 30 h, cells were stained with Hoescht33342 at a final concentration of 2 μg/mL in cell media for 30 min at 37 °C and 5% CO_2_. The plate was placed on an IN Cell 6000 Analyzer (GE Healthcare) with a heated stage and 50 fields of view were imaged under 4′,6-diamidino-2-phenylindole (DAPI) and FITC channels at ×60 magnification (Nikon ×60/0.95, Plan Apo, Corr Collar 0.11–0.23, CFI/60 lambda). Images were exported as TIFF files for downstream analysis. DnaJB8–mClover3, mClover3, and WT HEK293 cells were plated and imaged in triplicates. Total cell counting was done using the CellProfiler v3.0 software^50^ by selecting for DAPI (total cells in acquired images) and mClover3 (total expressing cells in acquired images). Puncta-containing cells were counted manually by two different observers and the data were reported as the average with a standard deviation between both observers. Expression of Clover and DnaJB8–Clover in 1× and 3× cell lines was quantified from Western blot analysis and by fluorescence intensity of Clover quantified using ImageJ^51^.

### Recombinant expression and purification of DnaJB8 and DnaJB8ΔCTD

The vector used for DnaJB8 expression was a pET-29b vector containing the gene for human DnaJB8, a T7 promoter to activate DnaJB8 expression, a His-tag region at the end of the gene, and a gene for kanamycin resistance. The DnaJB8ΔCTD fragment was cloned into pET-29b using Gibson assembly. The primers used are listed in Supplementary Table 2. The same protocol was used to express and purify the WT DnaJB8 and DnaJB8ΔCTD proteins. The vector constructs were transformed into *Escherichia coli* BL-21 (DE3) cells and plated onto 2× LB plates containing 0.05 mg/mL kanamycin. Twelve milliliters of 2× LB and 0.05 mg/mL kanamycin were prepared and inoculated with a single colony from the plate. This small culture was incubated overnight at 37 °C while shaking at 220 r.p.m. In the morning, the 12 mL culture was added to 1 L of 2× LB supplemented with 0.05 mg/mL kanamycin and incubated at 37 °C while shaking at 220 r.p.m. Once OD600 reached 0.6–0.8 AU, 1 mL of 1 M IPTG was added to induce DnaJB8 expression. After incubation for an additional 4 h, the cells were harvested by spinning down the culture at 4000 *g* for 20 min. The resulting cell pellet was resuspended in 25 mL 1× PBS and 1 mM PMSF in preparation for insoluble fraction separation. The resuspended cells were sonicated at 30% power, 5× pulse for 10 min using an Omni Sonic Ruptor 4000 (Omni International). The lysed cells were pelleted at 10,000 × *g* for 30 min and the supernatant was discarded. The insoluble pellet was rinsed with 1× PBS, 0.75% Tween-20, and again pelleted at 10,000 × *g* for 30 min.

The insoluble cell pellet was resuspended in 50 mL lysis buffer (8 M guanidinium HCl, 50 mM HEPES, 20 mM imidazole, 1 mM DTT pH 7.5) and sonicated at 30% power, 3× pulse for 1 min to solubilize the DnaJB8 from the insoluble pellet. After a 30-min incubation at room temperature, the cellular debris was pelleted at 15,000 × *g* for 30 min. The resulting supernatant was mixed with 2 mL HisPur^TM^ Ni-NTA Resin (Thermo Scientific) for 1 h before being loaded onto a gravity column. The column was washed with an additional 50 mL of lysis buffer, followed by 50 mL of a second wash buffer (50 mM HEPES, 20 mM imidazole, 1 mM DTT pH 7.5 in H_2_O). The protein was eluted with 30 mL of elution buffer (50 mM HEPES, 500 mM imidazole, 1 mM DTT pH 7.5 in H_2_O) and collected in 2 mL fractions. After selecting for fractions with high purity, the DnaJB8 solution was loaded into 3.5 kDa cutoff Biotech CE Dialysis Tubing (Spectrum Labs) and dialyzed overnight at 4 °C in 50 mM ammonium formate to minimize assembly. The protein was then lyophilized and stored at −80 °C for future use.

### Dynamic light scattering

All samples were prepared at 1.2 mg/mL in 1× PBS, 1 mM DTT pH 7.4. All protein samples were filtered through a 0.22-μm PES sterile filter and loaded in triplicate onto a 384-well clear flat-bottom plate. The plate was loaded into a Wyatt DynaPro Plate Reader III and set to run continuously at room temperature at a scanning rate of 1 scan/15 min, with 1 scan composed of ten acquisitions. The data were analyzed using the Wyatt Dynamics software version 7.8.2.18. Light-scattering results were filtered by the sum of squares (SOS) < 20 to eliminate statistical outlier acquisitions within each scan. For DnaJB8 in 1× PBS (150 mM) buffer, one of the triplicates contains partial data due to high SOS values. This is a result of increasing polydispersity and heterogeneity, which is consistent with oligomers of that size. *R*_h_ of observed particles for three time points (0, 10, and 20 h) was reported as histograms as a function of mass%. The mass% contribution of smaller particles in the full-length DnaJB8 runs in 1× PBS (150 mM) and (285 mM) buffer was reported as a function of mass% over time using the SOS filter and a size filter of <10 nm. Data for DnaJB8_F→S_, JD_1–82_, and CTD_170–232_ was reported as a function of *R*_h_ over time with the SOS filter applied.

### Modeling of full-length DnaJB8 using Rosetta and XL–MS restraints

Given the globular conformations of the JD and CTD, we considered how the cross-links identified for the full-length protein could guide the JD–CTD interaction. Using Rosetta we assembled a monomeric conformation leveraging the JD and CTD conformations while keeping the G/F and S/T regions fully expanded. This starting model was then used in a relax protocol in conjunction with cross-links (Supplementary Data 1) as constraints to produce an ensemble of 1000 collapsed conformations. A representative low scoring model was selected for further analysis. For acid–acid and acid–lysine contacts 21 and 16 Å distance thresholds were used as restraints. The HYDROPRO^52^ software was used to calculate radii of hydration from structural models.

### Conservation mapping

DnaJB8 homolog sequences were identified using Blast^53,54^ and the sequences were aligned using Clustal Omega^55^. The protein sequence alignment and structure of DnaJB8 JD (PDB ID: 2DMX) were used as input in Al2Co^56^ to map the conservation onto the structural models. The conservation was mapped onto the models in PyMOL.

### Coevolutionary variation analysis

The GREMLIN software^31–33^ was used to identify covarying amino acid pairs from a DnaJB8 protein sequence alignment. A probability of 0.7 was used to threshold the data to identify amino acids with a strong coupling.

### Solid-state NMR analysis

DnaJB8 was uniformly labeled with ^13^C and ^15^N (U-^13^C, ^15^N) and grown in M9-Minimal media supplemented with U-^13^C glucose (Isotec) and ^15^N NH_4_-Cl (CIL) and purified using the same protocol as the unlabeled form. Chaperone oligomers were prepared in PBS buffer with different NaCl concentrations. Lyophilized DnaJB8 was resuspended in 1 mL of PBS buffer in which the final NaCl concentration was 100 or 285 mM, respectively, for the two samples measured by MAS NMR. Each sample was packed into a 3.2-mm MAS NMR rotor (Bruker Biospin) by sedimentation^57^, using a device that permits the one-step centrifugation of the sample suspension directly into the NMR sample holder. For this, we employed a specifically designed device for use in a swinging-bucket SW 32 Ti ultracentrifuge rotor from Beckman Coulter (Indianapolis, IN)^57^. The empty MAS NMR sample holder was inserted into the bottom of the sedimentation device and the protein suspension was pipetted into the device’s funnel, followed by centrifugation at 175,000 × *g* in an Optima L-100 XP Ultracentrifuge for 1 h. Subsequently, excess of the supernatant fluid was removed. Sample tubes were washed with another 1 mL buffer solution, after which a second packing step using the same parameters was performed. Finally, the supernatant was removed, spacers were placed on the top of the hydrated sedimented protein oligomers, and rotors were closed with the drive cap, and sealed with a small amount of epoxy to avoid sample dehydration.

Experiments were performed on Bruker 600 and 750 MHz spectrometers at 277 K temperature using triple-channel (HCN) 3.2-mm MAS EFree probes. All experiments were done using two-pulse phase-modulated^58^ proton decoupling of 83 kHz during acquisition. SPE measurements were performed using a 6 μs 90° pulse on ^13^C, 3 s recycle delay, 1k scans, and 1D CP experiments were performed using a 3.1 μs 90° ^1^H pulse, 2 ms contact time, recycle delay of 3 s, and 1k scans. 1D refocused INEPT experiments were done using 3 μs 90° proton pulse, 4 μs 90° pulse on ^13^C, recycle delay of 2 s, and 1k scans. The ^13^C-^13^C 2D CP-DARR experiments were performed using 25 ms mixing time, 1 ms contact time, 3.1 μs 90° proton pulse, 6 μs 90° pulse on ^13^C, recycle delay of 2.8 s, and 128 scans per t_1_ point. The 2D ^15^N-^13^Cα experiment was performed using 3 μs 90° proton pulse, 900 μs and 3.25 ms ^1^H-^13^C and ^13^C-^15^N contact times, respectively, 8 μs 180° pulse on ^15^N, recycle delay of 2 s, and 576 scans per t_1_ point. The amino acid type and secondary structure were predicted using the PLUQ program^40^ applied to the chemical shifts in the 2D ^13^C-^13^C spectrum. Linewidth analysis was done using the UCSF Sparky NMR analysis program^59^. Spectral acquisitions were done with the Bruker Topspin software and processing was done with the NMRpipe v10.9, CCPNMR v2.4, and Sparky v3.115 software packages^59–62^.

### Simulations and synthetic NMR spectra

The structure of the DnaJB8 JD in solution was determined previously using solution NMR^44^, allowing us also to generate a synthetic ^13^C-^13^C 2D spectrum using the corresponding solution NMR chemical shifts from the BMRB (entry 11417). To simulate approximate 2D NMR spectra of the other three domains, we made use of the results of MD simulations of full-length DnaJB8. The starting DnaJB8 conformation was produced using ROSETTA v3.12 with a fully expanded conformation of the G/F and S/T domains while keeping the JD and CTD in the folded conformations. MD simulations were prepared using Maestro^63,64^ and carried out in Desmond running the amber99 forcefield. Estimated chemical shifts of the resulting structural models were generated using the SPARTA+ v2.9 package^41^.

### Recombinant expression and purification of DnaJB8_F→S_, JD, and CTD

Both vector constructs containing DnaJB8 JD and CTD, respectively, were cloned into pET-29b using Gibson assembly. The primers used are listed in Supplementary Table 2. The DnaJB8_F→S_ construct was purchased from Genscript and cloned into pET-29b. These vectors were transformed into *E. coli* BL-21 (DE3) cells and plated onto 2× LB plates with 0.05 mg/mL kanamycin. Twelve milliliters of 2× LB with 0.05 mg/mL kanamycin were prepared and inoculated with a single colony from each plate. These small cultures were incubated overnight at 37 °C while shaking at 220 r.p.m. In the morning, the 12 mL culture was added to 1 L of 2× LB supplemented with 0.05 mg/mL kanamycin and incubated at 37 °C while shaking at 220 r.p.m. Once OD600 reached 0.6–0.8 AU, 1 mL of 1 M IPTG was added to induce protein expression. After incubation for an additional 4 h, the cells were harvested by spinning down the culture at 4000 × *g* for 20 min. For preparing ^15^N JD, a single colony was inoculated into 10 mL 2× LB supplemented with 0.05 mg/mL kanamycin and incubated for 7–8 h at 37 °C while shaking at 220 r.p.m. The 10 mL culture was then mixed into 100 mL of M9 minimal media (42 mM Na_2_HPO_4_, 22 mM KH_2_PO_4_, 8.5 mM NaCl, 0.1 mM CaCl, 2 mM Mg_2_SO_4_, 1E-4% thiamine, 0.4% glucose, 187 mM NH_4_Cl, 0.05 mg/mL kanamycin) and incubated overnight at 37 °C while shaking at 220 r.p.m. In the morning of the following day, the cells were spun down at 2000 × *g* for 10 min and resuspended in 20 mL of M9 minimal media containing ^15^N-labeled NH_4_Cl in place of the unlabeled molecule. This was immediately added to 1 L of M9 minimal media with ^15^N-labeled NH_4_Cl and allowed to incubate at 37 °C while shaking at 220 r.p.m. Once OD600 reached 0.6–0.8 AU, 1 mL of 1 M IPTG was added to induce protein expression. After incubation for an additional 4 h, the cells were harvested by spinning down the culture at 4000 × *g* for 20 min. The cell pellets were resuspended in 20 mL of soluble wash buffer (SWB) (50 mM KPO_4_, 300 mM NaCl, 10% glycerol, 1 mM PMSF, 10 mM BME, pH 8) and sonicated at 30% power, 5× pulse for 10 min using an Omni Sonic Ruptor 4000 (Omni International). After incubation at room temperature for 1 h, the cell lysate was spun down at 15,000 × *g* for 30 min to separate the soluble supernatant from the insoluble pellet. The supernatant was mixed with 2 mL TALON® Metal Affinity Resin (Clontech) and incubated while shaking at 4 °C for 1 h. The protein–resin slurry was loaded onto a gravity column and washed with an additional 40 mL of SWB. This was followed by subsequent washes: 20 mL SWB with 0.5% Triton X-100, 20 mL SWB with adjusted 700 mM NaCl, 20 mL SWB with 0.1 mM ATP and 5 mM MgCl_2_, and an additional 40 mL of SWB. The protein was eluted with 16 mL SWB with 200 mM Imidazole into 2 mL fractions. After selecting for fractions with high purity, the protein solution was loaded into 3.5 kDa cutoff Biotech CE Dialysis Tubing (Spectrum Labs) and dialyzed overnight at 4 °C in 1× PBS to restore native folding. Both domain constructs were further enriched by running on a GE Superdex 200 Increase 10/300 column in 1× PBS 1 mM DTT pH 7. The protein was aliquoted and flash frozen in liquid nitrogen and stored at −80 °C for future use.

### SEC-MALS

DnaJB8_F→S_, JD_1–82_, and CTD_170–232_ constructs at a concentration of 5.1, 5.0, and 6.6 mg/mL, respectively, in 1× PBS were filtered through a 0.1-μm filter to remove larger impurities. Each sample was further filtered using a 0.22-μm centrifugal filter before 100 μL was applied to a Superdex 200 Increase 10/300 column equilibrated in 1× PBS with 1 mM TCEP. The column was in line with a Shimadzu UV detector, a Wyatt TREOS II light-scattering detector, and a Wyatt Optilab tREX differential-refractive-index detector. The flow rate was 0.5 mL/min. The data were analyzed with Wyatt’s ASTRA software version 7.1.0.29. SEDFIT^65^ was used to calculate the dn/dc of the protein.

### Circular dichroism

Recombinant CTD_170–232_ domain constructs were transferred into 10 mM NaPO_4_, 150 mM NaF, pH 7.4 buffer at a concentration of 40 μM. The experiment was run using a Jasco J-815 Circular Dichroism instrument with a PMT (photomultiplier tube) detector using a 10-mm quartz cuvette. Six accumulations were taken at a speed of 50 nm/min along the ultraviolet spectrum from 190 to 300 nm. Spectra analysis was done using the BeStSel online software^66,67^ to determine secondary structural composition.

### CTD model generation

Fragment libraries for the CTD sequence were generated using the Robetta server. Five thousand models were produced using the ab initio protocol and clustered to identify unique conformations. The lowest scoring models from the top clusters showed high structural similarity. The identified cross-links from the CTD in full-length DnaJB8 or isolated CTD were evaluated for consistency with each model in the ensemble. For acid–acid and acid–lysine cross-links a distance threshold of 21 and 16 Å, respectively, was considered as satisfied and consistent with the chemistry. Distances were calculated using a custom script in MATLAB ver.R2019a and the cross-link pairs were visualized using PyMOL. The HYDROPRO^52^ software was used to calculate radii of hydration from structural models.

### Fluorescence polarization

JD_1–82_ was labeled with 10× FITC-maleimide (Sigma) in 1× PBS 1 mM TCEP for 2 h at room temperature. The reaction was quenched and the excess dye was removed using a Superdex 75 Increase 10/300 GL size-exclusion column (GE). For all experiments, 0.2 μM JD_1–82_ was incubated in triplicate with a titration gradient of Hsp70 (150–0 μM) or CTD_170–232_ (150–0 μM) in 1× PBS, 1 mM TCEP, pH 7.4. The JD–CTD experiment was performed as three technical replicates, each in triplicate. For competition experiments, labeled JD_1–82_ was mixed with 3.125 μM CTD in triplicate and incubated at room temperature for 1 h before adding a titration gradient of Hsp70 (150–0 μM). FP readings were taken with excitation at 494 nm and emission at 525 nm. The data were fit to a one site-specific binding model using GraphPad Prism 7.04.

### Solution NMR with 15N-labeled JD and CTD

The ^15^N-labeled JD and CTD were exchanged into 20 mM Tris 100 mM NaCl 1 mM DTT pH 7 buffer using a Superdex 75 Increase 10/300 GL size-exclusion column (GE) in preparation for solution NMR. Each HSQC (heteronuclear single quantum coherence) run was performed with 50 μM ^5^N-labeled JD for 4 h at 1 scans/min with the temperature fixed at 299 K. After each run, unlabeled CTD was titrated into the sample at 2× (100 μM) the JD concentration, following serial dilutions of 1× (50 μM), 0.5× (25 μM), 0.25× (12.5 μM), and 0.125× (6.125 μM) sequentially. All scans were collected on an Agilent DD2 600 MHz instrument outfitted with a cold probe at the UT Southwestern Biomolecular NMR Facility. Each spectrum was converted into a readable format and phase corrected using NMRPipe^60^. Peak assignments were based on the deposited information from BMRB (11417). The software Sparky^59,61^ was used to analyze the peak shifts across all spectra.

### Recombinant expression and purification of HspA1A

*HspA1A* gene was cloned into the pMCSG7 plasmid^68^ and transformed into BL-21 (DE3) cells and plated onto 2× LB plates with 0.1 mg/mL ampicillin. Twelve milliliters of 2× LB with 0.1 mg/mL ampicillin were prepared and inoculated with a single colony from each plate. These small cultures were incubated overnight at 37 °C while shaking at 220 r.p.m. In the morning, the 12 mL culture was added to 1 L of 2× LB supplemented with 0.1 mg/mL ampicillin and incubated at 37 °C while shaking at 220 r.p.m. Once OD600 reached 0.6–0.8 AU, 1 mL of 1 M IPTG was added to induce protein expression. The cells continued to incubate overnight at 12 °C while shaking at 220 r.p.m. After incubation, the cells were lysed using a PandaPlus 2000 homogenizer (GEA) by pressing the cells with 10,000 p.p.m. pressure. The lysate was spun at 15,000 × *g* for 45 min to remove insoluble cell components, and the resulting supernatant was mixed with 2 mL TALON® Metal Affinity Resin (Clontech) and incubated at 4 °C for 1 h. The slurry was spun down at 700 × *g* for 2 min to remove the majority of the buffer and the beads were added onto a gravity column. The beads were washed with 6 CV (column volumes) of wash buffer (50 mM Tris, 500 mM NaCl, 10 mM imidazole, 5 mM β-mercaptoethanol (βME), pH 8) and eluted with 5 mL of elution buffer (50 mM Tris, 500 mM NaCl, 300 mM imidazole, 5 mM βME, pH 8). HspA1A-containing fractions were confirmed by SDS-PAGE and pooled together for desalting. Desalting/buffer exchange was performed using a PD-10 desalting column (GE Healthcare), where HspA1A fractions were transferred into anion-exchange wash buffer (50 mM Tris, 20 mM NaCl, 1 mM DTT, pH 8.75). The protein was loaded onto a HiTrap Q HP anion-exchange column (GE Healthcare) and eluted across a gradient of anion-exchange elution buffer (50 mM Tris, 1 M NaCl, 1 mM DTT, pH 8.75). HspA1A-containing fractions were once again combined and loaded onto a Superdex™ 200 Increase 10/300 GL (GE Life Sciences) size-exclusion column, where HspA1A was further purified and transferred into 1× PBS, 1 mM DTT, pH 7.4 buffer for all subsequent experiments.

### Reporting summary

Further information on research design is available in the Nature Research Reporting Summary linked to this article.

## Supplementary information

Supplementary Information

Description of Additional Supplementary Files

Supplementary Data 1

Supplementary Data 2

Reporting Summary

## Data Availability

Raw cross-linking mass spectrometry data are available in Supplementary Data 1. Raw DLS data are available in Supplementary Data 2. Other supporting data are available upon request from the authors. Publicly available data used in this study include: x-ray structure of the DnaJB8 J-domain (PDB ID: 2DMX), HSQC peak assignments for the DnaJB8 J-domain (BMRB: 11417), x-ray structure of DnaK in complex with DnaJ (PDB ID: 5NRO) and the nmr model of DnaJB6bΔS/T (PDB ID: 6U3R). Source data are provided with this paper.

## Source data

Source Data
