# Supplementary material for: Regulatory inter-domain interactions influence Hsp70 recruitment to the DnaJB8 chaperone
Source: Nat Commun. 2021 Feb 11;12:946. doi: 10.1038/s41467-021-21147-x (PMC7878476; doi:10.1038/s41467-021-21147-x)
Supplement: Supplementary file 1 — Supplementary Information [file 41467_2021_21147_MOESM1_ESM.pdf]

SUPPLEMENTARY INFORMATION

Regulatory inter-domain interactions influence Hsp70 recruitment to the DnaJB8 chaperone

Bryan Ryder, Irina Matlahov, Sofia Bali, Jaime Vaquer-Alicea, Patrick C.A. van der Wel, Lukasz A. Joachimiak

Supplementary Data

Supplementary Data 1. Primary XL-MS data for DnaJB8 and its domains.

Supplementary Data 2. Primary DLS measurements for DnaJB8 oligomers.

Supplementary Tables

Supplementary Table 1. Residue distribution per domain with net charge for each domain.

Domain aa. length	Domain	Residue																				Net charge for the domain
		A	R	N	D	Q	E	G	H	I	L	K	M	F	P	S	T	Y	V	W	C	
**1-75**	**JD**	8	5	3	6	1	7	2	1	1	6	10	1	1	3	7	0	5	4	2	1	+2
**76-148**	**G/F**	6	5	2	5	0	5	13	2	1	2	0	1	14	6	6	3	2	0	1	0	-5
**149-185**	**S/T**	0	0	1	0	0	1	7	1	0	1	1	3	4	0	15	4	0	1	0	1	0
**186-232**	**CTD**	0	2	3	2	3	6	4	7	2	2	6	1	0	0	2	3	0	6	1	0	0

Supplementary Table 2. Primers used for cloning DnaJB8 variants.

Primer Name	Sequence
DnaJB8_FM5_for	5'-GAACCGTCAGATCCGCTAGCGCCGCCACCATGGCTAACTACTACGAAGTGCTGGGCGTG-3'
DnaJB8_FM5_rev	5'-GCCGGATCCAGCAGCGGAGCCAGCAGAACCCTTGCTGTCCATCCATTTGAGCTGCTCC-3'
DnaJB8_pet29b_for	5'-ATAATTTTGTTTAACTTTAAGAAGGAGATATACATATGGCTAACTACTACGAAGTGCTGG-3'
DnaJB8_pet29b_rev	5'-GCAGCCGGATCTCAGTGGTGGTGGTGGTGGTGCTCGAGCTTGCTGTCCATCCATTTGAGC-3'
Jdomain_rev	5'-GATCTCAGTGGTGGTGGTGGTGGTGCTCGAGGGGCGTGCTGGCCCCGCCACCAGCCCGCC-3'
CTD_for	5'-AATTTTGTTTAACAAGAAGGAGATATACATATGGGCTCCAGTTCTGGCAGCTCGGGGTTC-3'
CTDdel_rev	5'-CCGGATCTCAGTGGTGGTGGTGGTGGTGCTCAGGAAATTCTCCAAAGCCTGCCGAG-3'

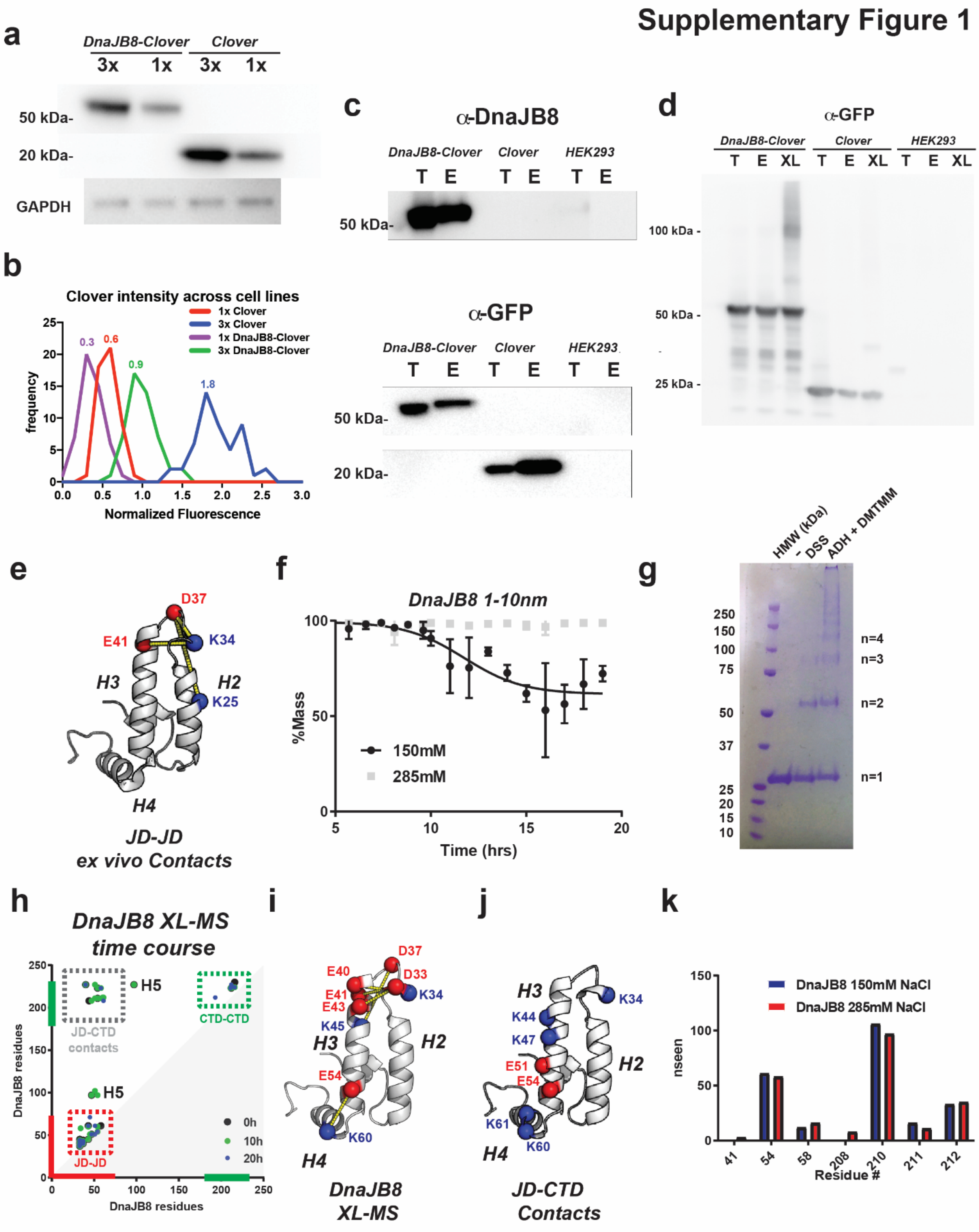
Supplementary Figure 1
a
DnaJB8-Clover
Clover
3x 1x 3x 1x
50 kDa-
20 kDa-
GAPDH
b
Clover intensity across cell lines
1x Clover
3x Clover
1x DnaJB8-Clover
3x DnaJB8-Clover
frequency
Normalized Fluorescence
c
α-DnaJB8
DnaJB8-Clover Clover HEK293
T E T E T E
50 kDa-
α-GFP
20 kDa-
d
α-GFP
T E XL T E XL T E XL
100 kDa -
50 kDa -
25 kDa -
e
D37
E41
K34
H3
H2
K25
H4
JD-JD
ex vivo Contacts
f
DnaJB8 1-10nm
%Mass
150mM
285mM
Time (hrs)
g
HMW (kDa)
- DSS
ADH + DMTMM
n=4
n=3
n=2
n=1
h
DnaJB8 XL-MS
time course
H5
JD-CTD
contacts
CTD-CTD
JD-JD
0h
10h
20h
DnaJB8 residues
i
D37
E40
D33
E41
E43
K34
K45
H3
H2
E54
K60
H4
DnaJB8
XL-MS
j
H3
K34
K44
K47
E51
E54
H2
K61
K60
H4
JD-CTD
Contacts
k
DnaJB8 150mM NaCl
DnaJB8 285mM NaCl
nseen
Residue #

Supplementary Figure 1. Biochemical and mass spectrometry analysis of DnaJB8 in cells and *in vitro*. (a) Western blot analysis of 1x DnaJB8-Clover, 3x DnaJB8-Clover, 1x Clover and 3x Clover cell lines to quantify levels of expression in each cell line. GAPDH western blot is shown as a loading control. The experiment was performed 3 independent times. (**b**) Normalized FITC intensity analysis of 1x Clover (red), 3x Clover (blue), 1x DnaJB8-Clover (purple) and 3x DnaJB8-Clover (green) cell lines. Intensity measurements were calculated from 50 images for each cell line and the signal normalized to DAPI fluorescence intensity. (**c**) Western blot analysis of input and anti-GFP nanobody elutions DnaJB8-Clover, Clover and HEK293 cell lines as detected with anti-DnaJB8 and anti-GFP antibodies. Total (T) and elutions (E) for each condition are shown. This experiment was performed 3 independent times. (**d**) Western blot analysis of DnaJB8-Clover and Clover proteins isolated from mammalian cell lines. Total (T), elutions (E) and crosslinked elutions (XL) of each condition are shown. Western blot was probed with GFP antibodies. This experiment was performed 3 independent times. **(e)** JD intra-domain ADH/DMTMM crosslinks identified from DnaJB8-Clover isolated from cells mapped onto the JD structure. JD is shown in cartoon representation and is colored in white. Sites of crosslink are shown as spheres and are colored red or blue for aspartic/glutamic and lysines, respectively. Dashed yellow lines connect linked amino acid pairs. **(f)** DnaJB8 particles with a R_h of 1-10nm in the DLS data were analyzed by proportion of the total sample (% mass) and binned by the size distribution of the constituent particles. The samples were run in triplicate with averages reported and error bars reporting the standard deviation. The time evolution of this mass fraction is shown for 150mM NaCl (black) and 285mM NaCl buffer conditions in 1xPBS. **(g)** SDS-PAGE coomassie gel showing DnaJB8 in the absence of crosslinker (left), with DSS (middle), and with DMTMM and ADH (right). **(h)** XL-MS contact map of DnaJB8 crosslinks identified using DMTMM and ADH from a time course: t=0hrs (large black dot), t=10hrs (medium green dot) and t=20hrs (small blue dot), small. The axes are colored in red and green for JD and CTD, respectively. Crosslink pairs between JD-CTD are shown in dashed box colored grey, red and green, respectively. Helix 5 crosslinks are denoted by H5. **(i)** JD intra-domain ADH/DMTMM *in vitro* crosslinks mapped onto the JD structure. JD is shown in cartoon representation and is colored in white. Sites of crosslink are shown as spheres and are colored red or blue for aspartic/glutamic and lysines, respectively. Dashed yellow lines connect linked amino acid pairs. **(j)** Seven cumulative crosslink sites identified from *in vitro* JD-CTD inter-domain ADH/DMTMM crosslinks mapped onto the JD structure. DnaJB8 JD is shown in cartoon representation and is colored in white. JD lysine sites that crosslink to CTD are shown as spheres and are colored blue and acids are colored red. **(k)** Histogram of frequency of ADH monolinks observed in normal and elevated ionic strength XLMS experiments.

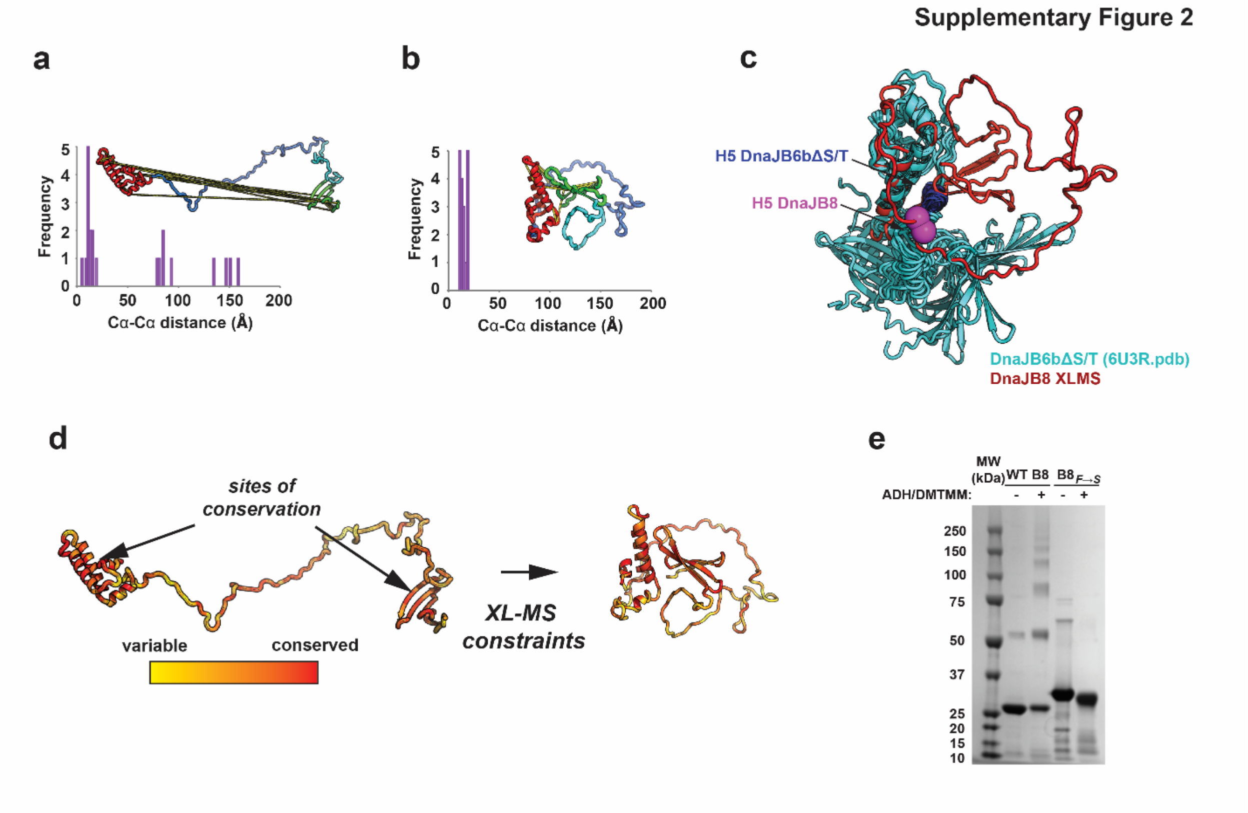

Supplementary Figure 2. Modeling the full length DnaJB8 monomer using crosslinks. (a) DnaJB8 ADH/DMTMM crosslinks mapped onto an expanded structure featuring the conserved JD and our predicted CTD model. The histogram of C_α-C_α distances shows 11 crosslinks that exceed the physical limitations for ADH/DMTMM. **(b)** Rosetta *ab initio* collapsed structure of DnaJB8 with ADH/DMTMM crosslinks mapped. The histogram of C_α-C_α distances shows that all crosslinks are satisfied when the JD and CTD are docked in this model. **(c)** Overlay of our Rosetta *ab initio* collapsed structure (red) with the solution NMR ensemble of the published DnaJB6bΔST deletion variant (cyan; PDBID: 6U3R) The proposed helix 5 (H5) is shown on the DnaJB8 (pink) and DnaJB6bΔST (blue) models. The position of H5 in our DnaJB8 XL-MS-constrained model is close to the position in the DnaJB6b model. The major difference is the position of the CTD, which is influenced by both the XL-MS constraints, and the inclusion of the S/T region that was deleted in the DnaJB6b construct. **(d)** Conserved surfaces on the JD and CTD in the full-length DnaJB8 model mediate the interaction. Highly conserved sites are colored in red (and highlighted by arrows) and variable positions are colored in yellow**.** Sequence-based conservation of DnaJB8 shows highly conserved faces along the JD, and some conserved faces in the CTD that overlap with the XL-MS identified surfaces. **(e)** SDS-PAGE coomassie gel of DnaJB8 and DnaJB8$_{F\rightarrow S}$ mutant crosslinked with ADH/DMTMM This experiment was performed 3 independent times.

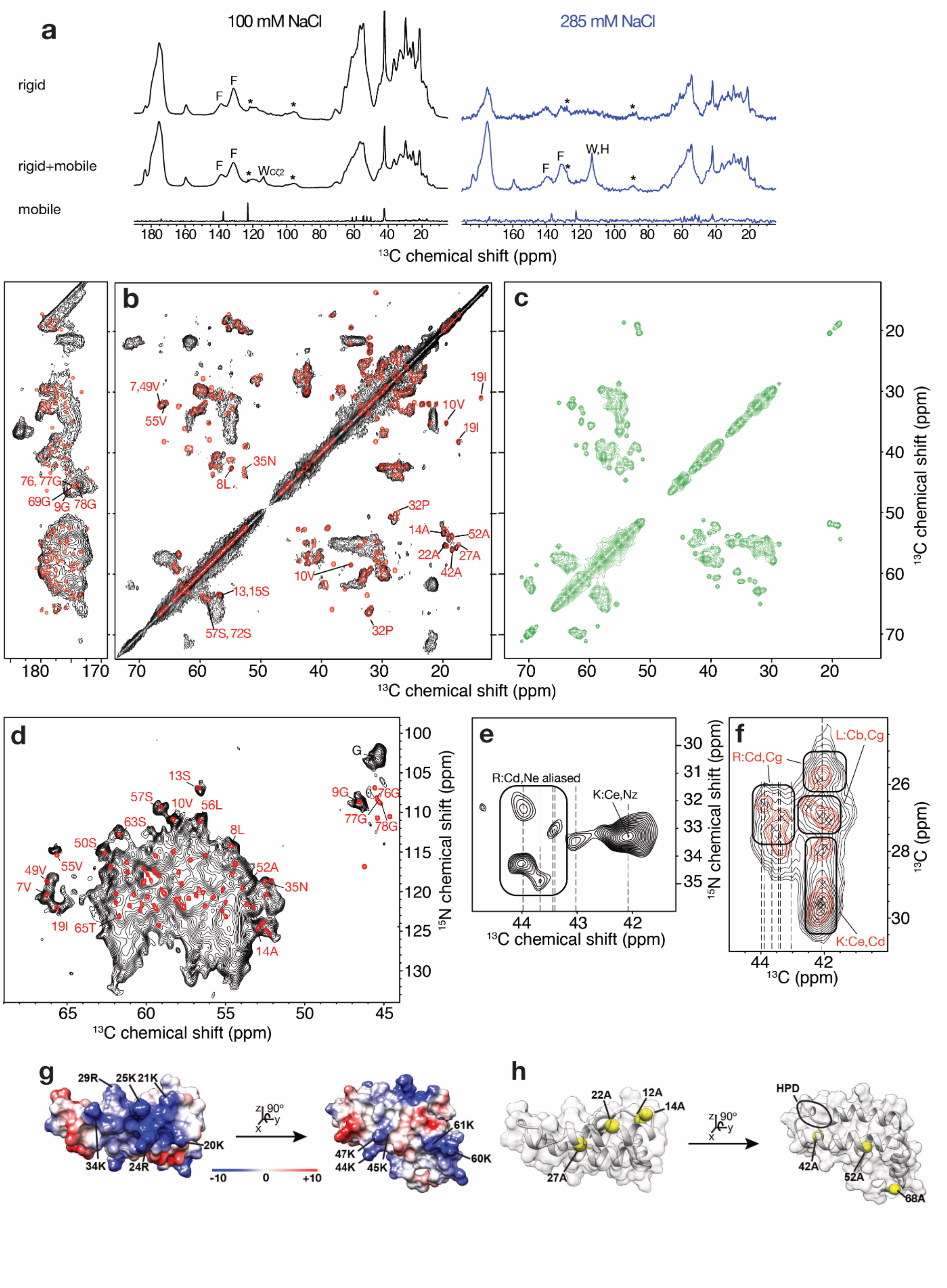
a
100 mM NaCl
285 mM NaCl
rigid
rigid+mobile
mobile
F
W,H
^{13}C chemical shift (ppm)
b
c
d
e
f
g
h
^{13}C chemical shift (ppm)
^{15}N chemical shift (ppm)
R:Cd,Ne aliased
K:Ce,Nz
R:Cd,Cg
L:Cb,Cg
K:Ce,Cd
^{13}C (ppm)
HPD

Supplementary Figure 3. Additional ssNMR data and analysis for DnaJB8 oligomers in PBS. (a) ^{13}C 1D spectra at 100mM NaCl (black) and elevated ionic strength (blue) that show rigid residues (CP), rigid and mobile residues (SPE) and only mobile residues (INEPT). **(b)** Overlay of the experimental (black) 2D ^{13}C-^{13}C CP-DARR spectrum of DnaJB8 oligomers uniformly labeled with ^{13}C and ^{15}N and synthetic (red) spectrum of the DnaJB8 JD in solution generated from BMRB chemical shifts (red assignments). **(c)** Simulated spectrum from three other domains (G/F, S/T and CTD), showing diagonal peaks and one-bond cross-peaks involving backbone Cα-Cβ and CO-Cα carbons. **(d)** 2D ^{13}C-^{15}N CP-based NCA experimental spectrum (black) of DnaJB8 oligomers uniformly labeled with ^{13}C and ^{15}N, overlaid with synthetic spectrum of the DnaJB8 JD in solution (red) from BMRB chemical shifts (red assignments). **(e)** 2D CP-based ^{13}C-^{15}N spectrum with Cε-Nζ and (aliased) Cδ-Nε correlations from immobilized Lysine and Arginine side chains, respectively. **(f)** Enlarged ^{13}C- C spectral region from panel (A) demonstrating good alignment of Arg, Lys and Leu signals between experimental ssNMR (black) and solution chemical shifts of the JD (red). Dashed lines mark known ^{13}C positions used to confirm amino acid types of signals. **(g)** Electrostatic surface potential of the DnaJB8 JD, with selected residues indicated. Highly positive and negative potentials are colored blue and red, respectively. White color represents neutral charge. **(h)** The same view showing the location of Ala with narrow peaks at 100 mM NaCl which disappear at 285 mM NaCl (Fig. 3, panel "c").

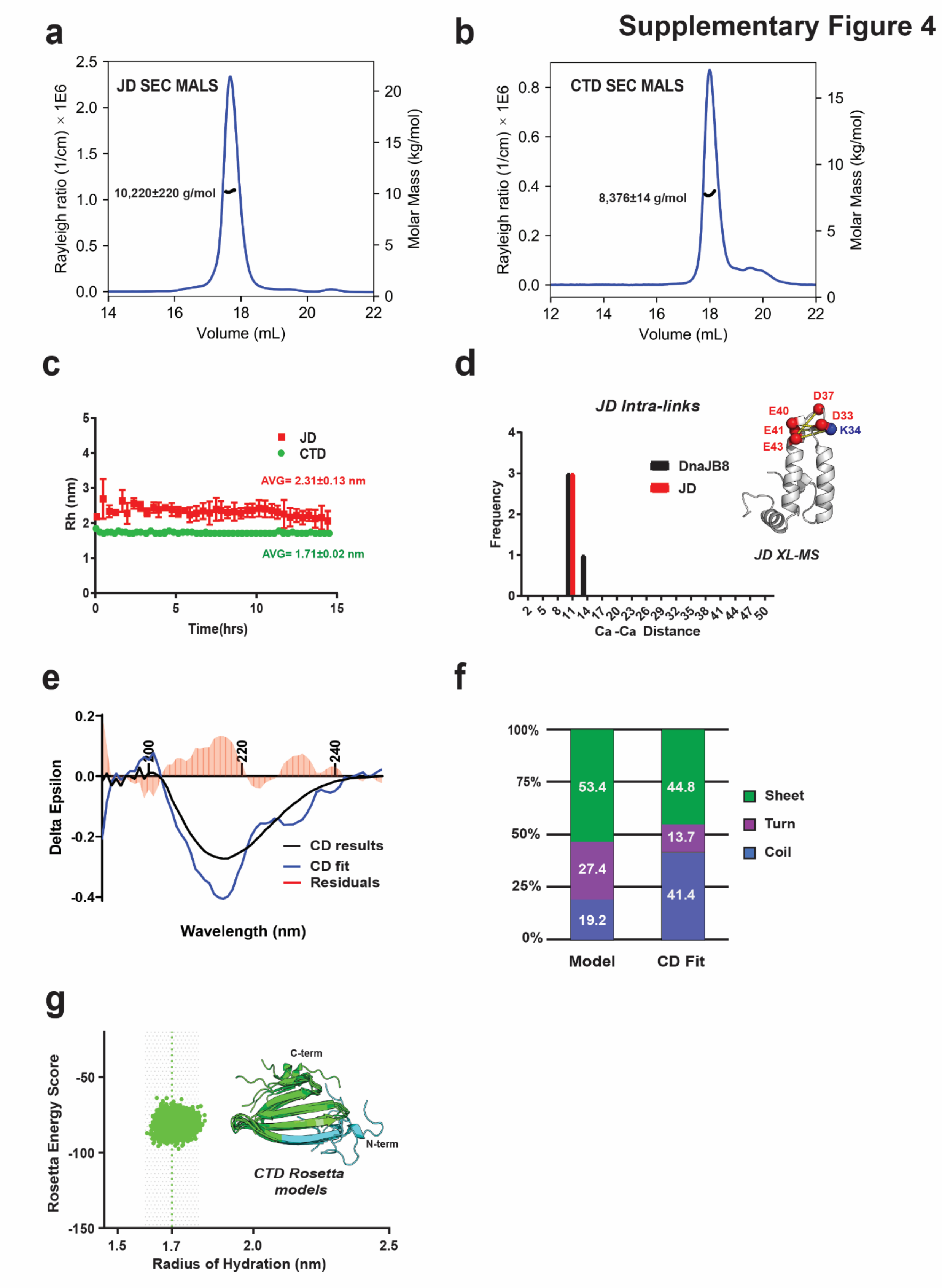

Supplementary Figure 4
a
JD SEC MALS
10,220±220 g/mol
Rayleigh ratio (1/cm) × 1E6
Molar Mass (kg/mol)
Volume (mL)
b
CTD SEC MALS
8,376±14 g/mol
Rayleigh ratio (1/cm) × 1E6
Molar Mass (kg/mol)
Volume (mL)
c
JD
CTD
AVG= 2.31±0.13 nm
AVG= 1.71±0.02 nm
Rh (nm)
Time(hrs)
d
JD Intra-links
DnaJB8
JD
Frequency
Ca -Ca Distance
D37
E40
D33
E41
K34
E43
JD XL-MS
e
Delta Epsilon
CD results
CD fit
Residuals
Wavelength (nm)
f
53.4
44.8
27.4
13.7
19.2
41.4
Sheet
Turn
Coil
Model
CD Fit
g
Rosetta Energy Score
C-term
N-term
CTD Rosetta models
Radius of Hydration (nm)

Supplementary Figure 4. JD and CTD are stable monomers. (a) SEC-MALS of $JD_{1\text{-}82}$ shows a single peak with a calculated molar mass of 10,220 ± 220 g/mol consistent with a monomer. **(b)** SEC-MALS of $CTD_{170\text{-}232}$ shows a single peak with a calculated molar mass of 8,376 ±14 g/mol consistent with a monomer. **(c)** DLS time course of $JD_{1\text{-}82}$ and $CTD_{170\text{-}232}$ constructs. Samples were run in triplicates with averages reported and error bars reporting the standard deviation. The average R_h of $JD_{1\text{-}82}$ and $CTD_{170\text{-}232}$ was calculated to be 2.30±0.12 nm and 1.71±0.02 nm, respectively. **(d)** Histogram of JD intra-domain crosslinks across XLMS experiments for DnaJB8(black), $JD_{1\text{-}82}$(red), and $JD_{1\text{-}82}$ mixed with $CTD_{170\text{-}232}$(grey). All intra-domain crosslinks satisfy the physical constraint of <20nm for the ADH/DMTMM crosslinkers. **(e,f)** CD spectra of CTD was analyzed using the Bestsel server. Secondary structure analysis of the data shows high β-sheet character (yellow) with some helix character (purple) and no random coil (blue). The results are compared to the CTD model generated from ab initio. **(g)** 5,000 Rosetta *ab initio* generated models of CTD. The structural models are consistent with R_h values derived from DLS (dashed line). Structural overlay of low energy scoring models reveals consistent pleated beta-sheet fold. Models are shown in cartoon representation and are colored in green.

Supplementary Figure 5

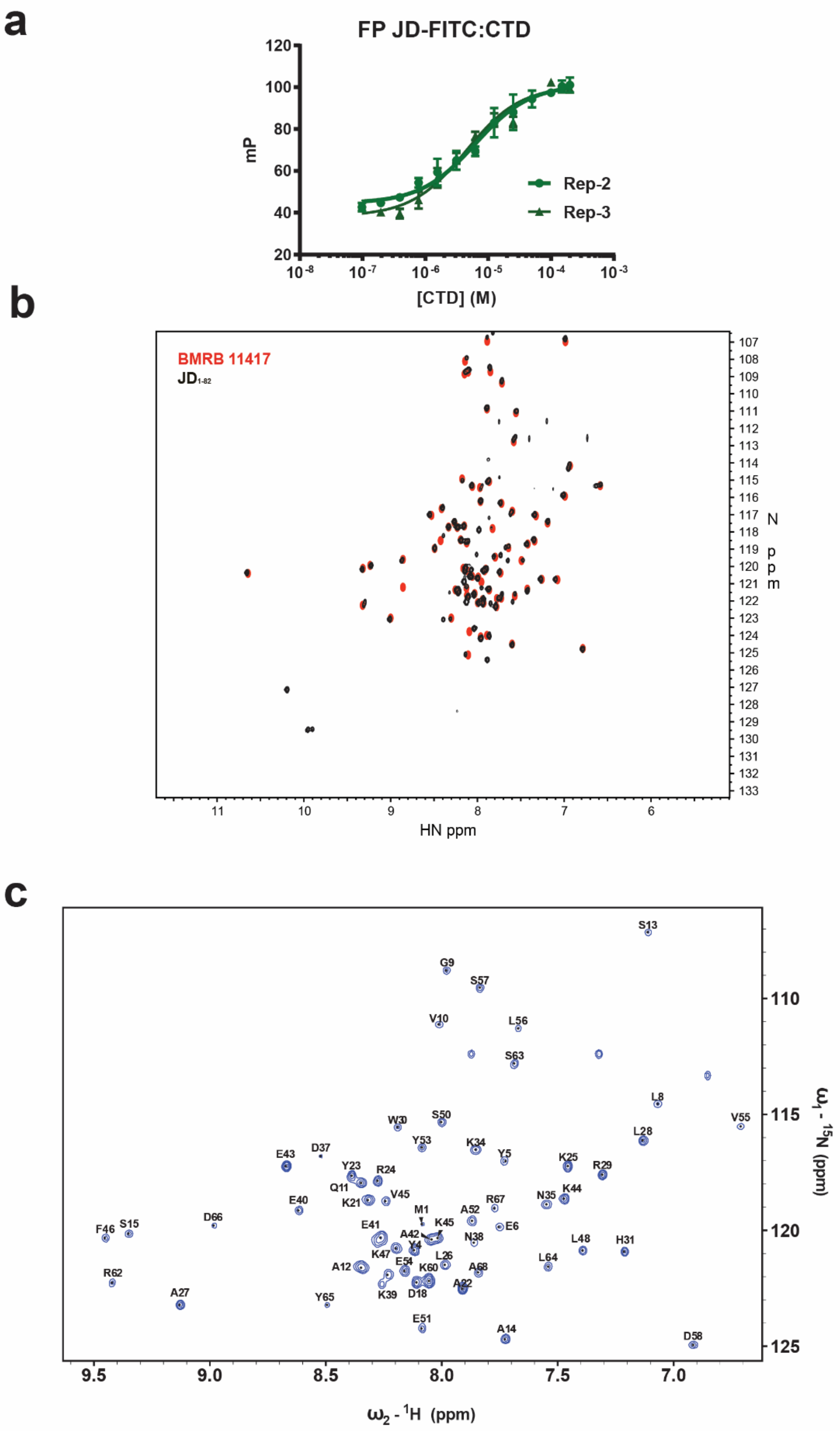

Supplementary Figure 5. JD_{1-82} construct spectra consistent with deposited chemical shifts. (a) Two technical replicates of the JD-FITC fluorescence polarization binding assay onto CTD as shown in Fig. 5a. Each replicate was performed in triplicate and shown as averages with standard deviations, yielding affinities of 6.21±0.94 µM and 4.43±0.55 µM, respectively. **(b)** Overlay of our ^{15}N-^{1}H HSQC spectra for JD_{1-82}(black) onto the submitted spectra for DnaJB8 JD (red) (BMRB:11417). 97% of assigned peaks in the published spectra were matched to our data. (**c)** Amino acid assignments mapped onto the HSQC spectra for the JD_{1-82} protein.

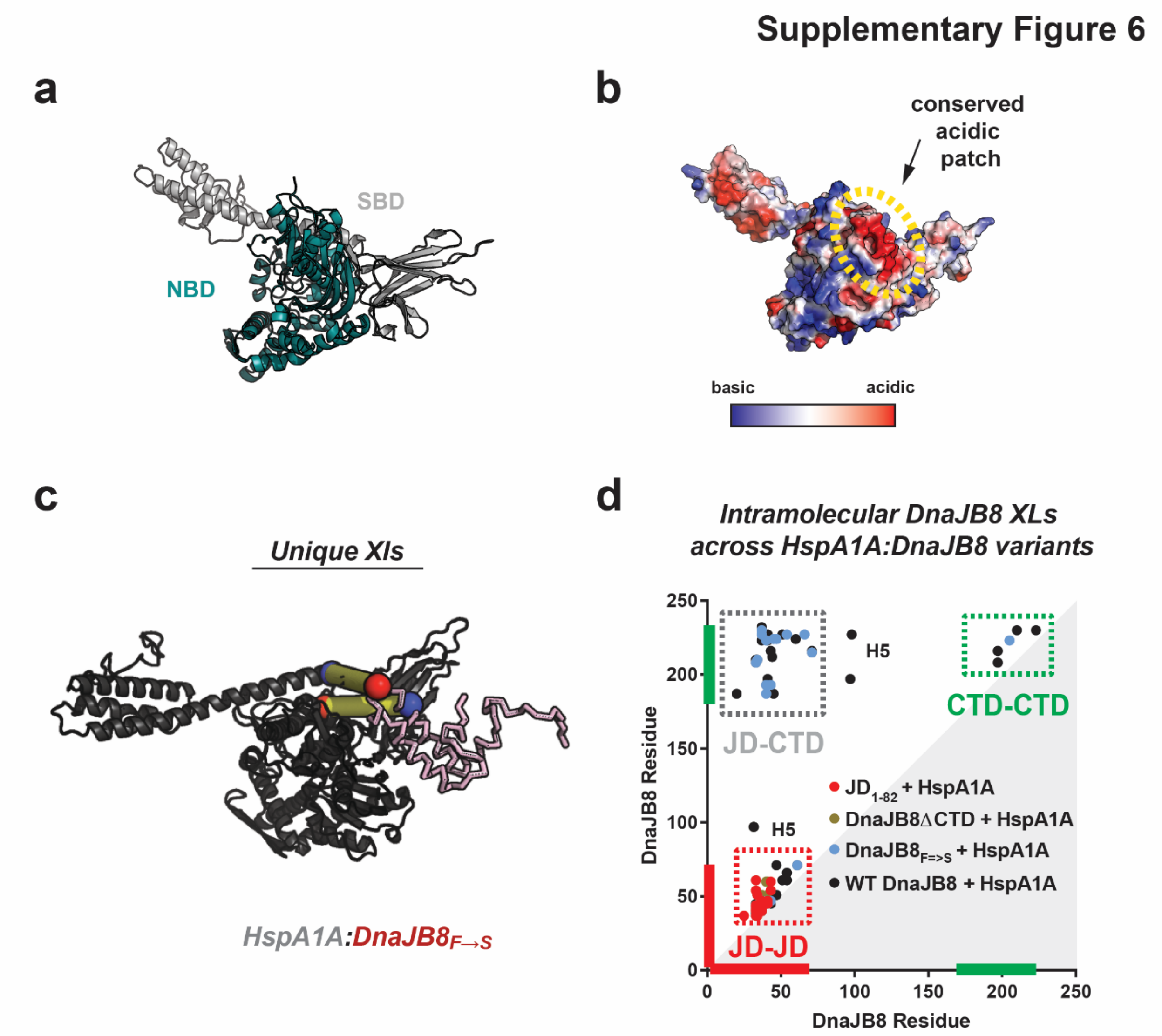

Supplementary Figure 6. Hsp70 proteins present a conserved acidic surface. (a) Structure of HspA1A colored by domain with the nucleotide binding domain (NBD) in turquoise and substrate binding domain (SBD) in grey. (**b)** Electrostatic surface mapped onto the HspA1A structure with the negatively charged Hsp40-JD-binding site circled in yellow. **(c)** The two intermolecular crosslinks identified across three XL-MS datasets between JD and HspA1A in the DnaJB8:HspA1A complex mapped onto JD-HspA1A model. JD is shown in ribbon representation and HspA1A in cartoon representation, colored pink and black respectively. Sites of crosslink are shown as red or blue spheres for aspartic/glutamic acid and lysines, respectively. Yellow lines connect linked amino acid pairs. **(d)** XL-MS contact map of changes in intramolecular DnaJB8 crosslinks identified using DMTMM and ADH in co-mixtures of different variants of DnaJB8 with HspA1A: WT DnaJB8:HspA1A (black), DnaJB8ΔCTD:HspA1A (brown), DnaJB8$_{F \rightarrow S}$:HspA1A (blue) and JD$_{1\text{-}82}$:HspA1A (red). The axes are colored in red and green for JD and CTD, respectively. Crosslink pairs between JD-CTD are shown in dashed box colored grey, red and green, respectively. Contacts to helix 5 in WT DnaJB8 are denoted with H5.
