# Supplementary material for: Regulatory inter-domain interactions influence Hsp70 recruitment to the DnaJB8 chaperone
Source: Nat Commun. 2021 Feb 11;12:946. doi: 10.1038/s41467-021-21147-x (PMC7878476; doi:10.1038/s41467-021-21147-x)
Supplement: Supplementary file 2 — Description of Additional Supplementary Files [file 41467_2021_21147_MOESM2_ESM.pdf]

Description of Additional Supplementary Files

Supplementary Data 1: Cross-linking mass spectrometry raw data

Supplementary Data 2: Dynamic light scattering raw data
