# Supplementary material for: Regulatory inter-domain interactions influence Hsp70 recruitment to the DnaJB8 chaperone
Source: Nat Commun. 2021 Feb 11;12:946. doi: 10.1038/s41467-021-21147-x (PMC7878476; doi:10.1038/s41467-021-21147-x)
Supplement: Supplementary file 5 — Reporting Summary [file 41467_2021_21147_MOESM5_ESM.pdf]

nature research

Corresponding author(s): PCA van der Wel, LA Joachimiak

Last updated by author(s): 2020/1/12

Reporting Summary

Nature Research wishes to improve the reproducibility of the work that we publish. This form provides structure for consistency and transparency in reporting. For further information on Nature Research policies, see Authors & Referees and the Editorial Policy Checklist .

Statistics

For all statistical analyses, confirm that the following items are present in the figure legend, table legend, main text, or Methods section.

n/a	Confirmed	
☐	☒	The exact sample size (*n*) for each experimental group/condition, given as a discrete number and unit of measurement
☐	☒	A statement on whether measurements were taken from distinct samples or whether the same sample was measured repeatedly
☒	☐	The statistical test(s) used AND whether they are one- or two-sided<br>*Only common tests should be described solely by name; describe more complex techniques in the Methods section.*
☒	☐	A description of all covariates tested
☒	☐	A description of any assumptions or corrections, such as tests of normality and adjustment for multiple comparisons
☐	☒	A full description of the statistical parameters including central tendency (e.g. means) or other basic estimates (e.g. regression coefficient) AND variation (e.g. standard deviation) or associated estimates of uncertainty (e.g. confidence intervals)
☒	☐	For null hypothesis testing, the test statistic (e.g. *F*, *t*, *r*) with confidence intervals, effect sizes, degrees of freedom and *P* value noted<br>*Give P values as exact values whenever suitable.*
☒	☐	For Bayesian analysis, information on the choice of priors and Markov chain Monte Carlo settings
☒	☐	For hierarchical and complex designs, identification of the appropriate level for tests and full reporting of outcomes
☒	☐	Estimates of effect sizes (e.g. Cohen's *d*, Pearson's *r*), indicating how they were calculated

Our web collection on statistics for biologists contains articles on many of the points above.

Software and code

Policy information about availability of computer code

Data collection	All DnaJB8 protein simulations were carried out with ROSETTA v3.12 (available at https://www.rosettacommons.org/). DnaJB8 molecular dynamics simulations were performed using Desmond release 2020-4 (available at https://www.deshawresearch.com/resources_desmond.html). Sequence analyses including net charge per residue were carried out with localCIDER v0.1.17 (https://pappulab.github.io/localCIDER/). NMR data was acquired using Bruker Topspin v3.5 (available at https://www.bruker.com/service/support-upgrades/software-downloads/nmr.html). Sequence covariance calculations were carried out using GREMLIN v2.01 (http://gremlin.bakerlab.org/). Conservation was calculated using Al2Co v1 (available at http://prodata.swmed.edu/download/pub/al2co/). Simulated spectra of DNAJB8 monomer was carried out using Sparta+ v2.9(available at https://spin.niddk.nih.gov/bax/software/SPARTA+/).
Data analysis	DnaJB8-mclover3 expressing HEK293 cell lines were analyzed with ImageJ v1.52p (available at: https://imagej.net/Downloads). All images of structures were produced in pymol v1.8.4.2. All plots were generated with GraphPad Prism v7.04. XL-MS data analysis was carried out using Xquest V2.1.3 and xProphet v2.5.5(available at http://proteomics.ethz.ch/cgi-bin/xquest2_cgi/index.cgi). Solution NMR data was processed using NMRpipe v10.9(available https://www.ibbr.umd.edu/nmrpipe/install.html) and analyzed SPARKY v3.115(available https://www.cgl.ucsf.edu/home/sparky/). Solid-state NMR data was processed using NMRPipe v10.9 (available at https://www.ibbr.umd.edu/nmrpipe/install.html) and analyzed using CCPNMR v2.4 (available at https://www.ccpn.ac.uk/v2-software/software/analysis). DLS data was analyzed using Dynamics software v7.8.2.18 (https://www.wyatt.com/products/software/dynamics.html). CD spectra were analyzed using BeSTsel test version(available at http://bestsel.elte.hu/). SEC-MALS data was analyzed using Astra v7.1.0.29 software (available at https://www.wyatt.com/products/software/astra.html). Radius of Hydration values were calculated using HYDROPRO v10 (available at http://leonardo.inf.um.es/macromol/programs/hydropro/hydropro.htm).

For manuscripts utilizing custom algorithms or software that are central to the research but not yet described in published literature, software must be made available to editors/reviewers. We strongly encourage code deposition in a community repository (e.g. GitHub). See the Nature Research guidelines for submitting code & software for further information.

Data

Policy information about availability of data

All manuscripts must include a data availability statement. This statement should provide the following information, where applicable:

- Accession codes, unique identifiers, or web links for publicly available datasets
- A list of figures that have associated raw data
- A description of any restrictions on data availability

Source data are provided with this paper. Raw crosslinking mass spectrometry data is available in Supplemental Data 1. Raw DLS data is available in Supplemental Data 2. Other supporting data available upon request from the authors. Publicly available data used in this study include: PDB structure of the DnaJB8 J-domain (PDB 2DMX) PDB structure of DnaK bound to DnaJ (PDB-5NRO) HSQC peak assignments for the DnaJB8 J-domain (BMRB-11417), and the structure of DnaJB6bΔS/T (PDB-6U3R).

Field-specific reporting

Please select the one below that is the best fit for your research. If you are not sure, read the appropriate sections before making your selection.

☒ Life sciences ☐ Behavioural & social sciences ☐ Ecological, evolutionary & environmental sciences

For a reference copy of the document with all sections, see nature.com/documents/nr-reporting-summary-flat.pdf

Life sciences study design

All studies must disclose on these points even when the disclosure is negative.

Sample size	We did not study populations either of animals or humans, thus population sample size is not applicable. In the case of Rosetta simulations, we chose 5000 as the number of models to build to for the CTD alone and 1000 for full-length DnaJB8 with XL-MS restraints. This is relatively standard in the field, balancing the size of the protein, access to cluster time and whether the simulations are restrained with experimental constraints. Using the available biohpc cluster these simulations represented ~2 weeks of computer time.
Data exclusions	No data were excluded in the analyses
Replication	Figure 1 and Supplementary Figure 1. Imaging of DnaJB8-Clover and Clover cells was performed as three biological replicates. Quantification of frequency of puncta was performed using CellProfiler in triplicate on at least 2,000 cells from populations of 300,000 cells, reported as averages with standard deviation. Western blot analysis of DnaJB8 Immunoprecipitation (IP) was performed two times, representative blot is shown. Western blot analysis of DnaJB8 IP (probed with anti-GFP and anti-DNAJB8 antibodies) was performed three times, representative blots are shown. DLS measurements for low and high salt were carried out twice as biological replicates with each experiment performed in triplicate. The data shown match experiments in Figure 3 but are partitioned according to two different bin sizes (1-10nm and 11-1000nm). Triplicate values for 1-10nm bin were plotted as averages with standard deviation. SDS-PAGE gel of crosslinked DnaJB8 was performed as three biological replicates. XL-MS on the crosslinked DnaJB8 was also performed two times, data shown matches the western blot. Crosslink experiments were performed three independent times. Raw XL-MS data is reported in Supplemental Data 1. DLS measurements for low and high salt were carried out twice as biological replicates with each experiment performed in triplicate. The data are shown as averages with standard deviation. Raw DLS data is reported in Supplementary Data 2.<br>Figure 2 and Supplementary Figure 2. 1000 DnaJB8 models were built using XL-MS restraints using a relax protocol in ROSETTA starting from an extended conformation. This sample size was chosen based on the protein size, and the given restraints from the XL-MS data in Figure 1 in order to optimize for time. A low energy conformation is shown that satisfies the XL-MS restraints. Crosslink positions are available in Supplemental Data 2. NPCR analysis was carried out once. Gremlin sequence analysis was performed once. NCPR calculations using localCIDER were performed once. Crosslink experiments for mutant DnaJB8 was performed three independent times. Raw XL-MS data is reported in Supplemental Data 1. DLS measurements for mutant DnaJB8 was carried out twice as biological replicates with each experiment performed in triplicate. The data are shown as averages with standard deviation. Raw DLS data is reported in Supplementary Data 2. SEC MALS data on mutant DnaJB8 was performed once. SDS-PAGE gel of crosslinked mutant DnaJB8 was performed as three biological replicates.<br>Figure 3 and Supplementary Figure 3. Solid state NMR experiments under low and high salt conditions were performed once for each of the two samples. ssNMR analysis was performed once each for low and high salt condition. Simulated spectra for DNAJB8 were calculated once.<br>Figure 4 and Supplementary Figure 4. SEC experiments on JD and CTD domains were performed as three biological replicates. Representative SEC chromatography profiles are shown for CTD, JD and standards. SEC MALS on JD and CTD were performed once. XL-MS analysis of CTD and JD experiments was carried as three biological replicates. A representative gel and XL-MS analysis are shown. Raw XL-MS data is available in Supplemental Data 2. 5000 models of CTD were built with ab initio ROSETTA. CTD SEC-MALS experiment was performed once. Sample size was increased for this experiment since the size of the protein construct is considerably smaller, allowing for more runs within a reasonable amount of time. DLS of JD and CTD samples was performed as three biological replicates with three technical replicates each. A representative dataset is plotted as averages with standard deviation. CD experiments were performed once using 6 scans. The data is plotted as an average of the 6 scans. XL-MS data match experiments reported in Figure 5.<br>Figure 5 and Supplementary Figure 5. Fluorescence polarization JD(FITC):CTD binding experiments were performed three times, each with three technical replicates. Data is shown as an average with standard deviation. XL-MS analysis of CTD:JD experiments was carried as three biological replicates. A representative XL-MS dataset is shown. Raw XL-MS data is available in Supplemental Data 2. Solution NMR experiments measuring chemical shifts between JD and CTD were performed once. Solution HSQC NMR experiments on 15N JD were performed once with each CTD concentration being a 2x dilution of each preceding sample starting with 2x and ending at 0.125x.<br>Figure 6 and Supplementary Figure 6. 1000 homology models of human HSPA1A were built with ROSETTA using the bacterial DnaK as a template (pdbid:5NRO). Sample size was chosen based on the same rationale as described above for Figure 2. Low energy conformation was used to produce the HSPA1A:JD(DNAJB8) complex using structural alignments to DnaK:DnaJ complex (pdbid:5NRO). HspA1A NCPR and

conservation calculations were performed once. Competition binding experiments were performed as two biological replicates with three technical replicates. Crosslink experiments between JD:HspA1A, WT DnaJB8:HspA1A, DnaJB8ΔCTD:JD and DnaJB8F=>S:JD were performed in triplicate and are reported in Supplementary Data 1.
Figure 7. N/A

Randomization	Samples were not allocated into groups, so randomization is not applicable.
Blinding	Samples were not allocated into groups; blinding was not applied.

Reporting for specific materials, systems and methods

We require information from authors about some types of materials, experimental systems and methods used in many studies. Here, indicate whether each material, system or method listed is relevant to your study. If you are not sure if a list item applies to your research, read the appropriate section before selecting a response.

Materials & experimental systems

n/a	Involved in the study
☐	☒ Antibodies
☐	☒ Eukaryotic cell lines
☒	☐ Palaeontology
☒	☐ Animals and other organisms
☒	☐ Human research participants
☒	☐ Clinical data

Methods

n/a	Involved in the study
☒	☐ ChIP-seq
☒	☐ Flow cytometry
☒	☐ MRI-based neuroimaging

Antibodies

Antibodies used	anti-GFP antibodies (Rockland; 600-401-215; 35460)<br>anti-DNAJB8 antibodies (abcam; ab235546; GR3229943-2)<br>Amersham ECL anti-Rabbit IgG Horseradish Peroxidase (cytiva; NA934-1ML; 17065618)
Validation	anti-GFP was validated via western blot by the manufacturer: Rockland (https://rockland-inc.com/store/Antibodies-to-GFP-and-Antibodies-to-RFP-600-401-215-O4L_18562.aspx)<br>anti-DnaJB8 was validated via western blot by the manufacturer: Abcam (https://www.abcam.com/dnajb8-antibody-ab235546.html)<br>anti-Rabbit IgG was validated via western blot by the manufacturer: Cytiva Life Sciences (https://www.cytivalifesciences.com/en/us/shop/protein-analysis/blotting-and-detection/blot[...]reagents/amersham-ecl-hrp-conjugated-antibodies-p-06260)

Eukaryotic cell lines

Policy information about cell lines

Cell line source(s)	Cell line 293T/17 [HEK 293T/17] (ATCC CRL-11268)
Authentication	Cell lines were not authenticated
Mycoplasma contamination	Cells were confirmed to be free of mycoplasma contamination
Commonly misidentified lines (See ICLAC register)	None were used in this study
